# GAS1 is required for NOTCH-dependent facilitation of SHH signaling in the ventral forebrain neuroepithelium

**DOI:** 10.1242/dev.200080

**Published:** 2021-11-11

**Authors:** Maike Marczenke, Daniele Yumi Sunaga-Franze, Oliver Popp, Irene W. Althaus, Sascha Sauer, Philipp Mertins, Annabel Christ, Benjamin L. Allen, Thomas E. Willnow

**Affiliations:** 1 Molecular Physiology, Max-Delbrueck-Center for Molecular Medicine, 13125 Berlin, Germany; 2 Department of Biology, Chemistry and Pharmacy, Freie Universitaet Berlin, 12169 Berlin, Germany; 3 Genomics Platform, Max-Delbrueck-Center for Molecular Medicine, 13125 Berlin, Germany; 4 Proteomics Platform, Max-Delbrueck-Center for Molecular Medicine, 13125 Berlin, Germany; 5 Department of Cell and Developmental Biology, University of Michigan Medical School, Ann Arbor, MI 48109, USA; 6 Department of Biomedicine, Aarhus University, 8000 Aarhus, Denmark

**Keywords:** Forebrain organizer region, Holoprosencephaly, NOTCH intracellular domain, Neuroepithelial precursor cells, HH co-receptors

## Abstract

Growth arrest-specific 1 (GAS1) acts as a co-receptor to patched 1, promoting sonic hedgehog (SHH) signaling in the developing nervous system. *GAS1* mutations in humans and animal models result in forebrain and craniofacial malformations, defects ascribed to a function for GAS1 in SHH signaling during early neurulation. Here, we confirm loss of SHH activity in the forebrain neuroepithelium in GAS1-deficient mice and in induced pluripotent stem cell-derived cell models of human neuroepithelial differentiation. However, our studies document that this defect can be attributed, at least in part, to a novel role for GAS1 in facilitating NOTCH signaling, which is essential to sustain a persistent SHH activity domain in the forebrain neuroepithelium. GAS1 directly binds NOTCH1, enhancing ligand-induced processing of the NOTCH1 intracellular domain, which drives NOTCH pathway activity in the developing forebrain. Our findings identify a unique role for GAS1 in integrating NOTCH and SHH signal reception in neuroepithelial cells, and they suggest that loss of GAS1-dependent NOTCH1 activation contributes to forebrain malformations in individuals carrying *GAS1* mutations.

## Abstract (precis)

**Summary:** GAS1 acts as co-receptor that integrates signaling by sonic hedgehog and NOTCH in neural progenitor cells, which are essential for controlling the differentiation of the forebrain neuroepithelium.

## INTRODUCTION

The mammalian forebrain develops from a simple neuroepithelial sheet at the anterior end of the neural plate: the anterior neuroectoderm. Several morphogen pathways provide instructive signals during early neurulation, including sonic hedgehog (SHH), which governs patterning processes along the dorso-ventral axis of the developing neural tube (reviewed by Dessaud et al., 2008). In the embryonic forebrain, SHH is initially produced from the prechordal plate (PrCP) at the anterior tip of the embryo. It acts on the overlying rostral diencephalon ventral midline (RDVM) to induce its own production and the expression of ventral forebrain markers. SHH transcriptional targets, such as NK2 homeobox 1 (NKX2.1), specify ventral midline identity and counteract dorsalizing signals by bone morphogenetic protein 4 (BMP4) (Hoch et al., 2009; Sousa and Fishell, 2010). In line with a prominent role for SHH in forebrain development, defects in SHH signaling in humans (Roessler et al., 1996) and in mouse models (Chiang et al., 1996) result in midline formation defects, ultimately causing craniofacial malformation and holoprosencephaly (HPE). HPE is the most frequent forebrain anomaly in humans and may include improper division of the forebrain hemispheres, as well as cyclopia and formation of a proboscis (Muenke and Beachy, 2000). Inheritable mutations in components of the SHH signaling pathway have been associated with human HPE, including mutations in *SHH*, in its receptor patched 1 (*PTCH1*), or in downstream transcription factors, such as GLI family zinc finger 2 (*GLI2*), SIX homeobox 3 (*SIX3*) and zinc-finger protein of the cerebellum 2 (*ZIC2*) (Roessler and Muenke, 2010).

Besides the canonical SHH receptor PTCH1, previous studies have identified additional cell surface proteins that facilitate SHH signal reception in the neuroepithelium and that cause midline malformations and HPE when mutated (reviewed by Christ et al., 2016). One of these auxiliary SHH receptors is growth arrest-specific 1 (GAS1), a 45 kDa glycoprotein attached to the plasma membrane via a glycosylphosphatidylinositol (GPI) anchor. Among other cell types, GAS1 is expressed in progenitor cells of the developing central nervous system (Allen et al., 2007; Lee and Fan, 2001). GAS1 facilitates interaction of SHH with PTCH1 under limiting SHH concentrations by forming co-receptor complexes, promoting SHH signaling in the cerebellum (Izzi et al., 2011) and spinal cord (Allen et al., 2011, 2007; Martinelli and Fan, 2007).

Loss of GAS1 in gene-targeted mice (Allen et al., 2007; Khonsari et al., 2013; Martinelli and Fan, 2007; Seppala et al., 2007, 2014) or in individuals with *GAS1* missense mutation (Pineda-Alvarez et al., 2012; Ribeiro et al., 2010) result in a range of craniofacial and forebrain malformations, including small eyes, cleft palate, fusion of nasal processes and HPE. These malformations are believed to originate from defects in early development of the rostral forebrain neuroepithelium, as judged from impaired expression of *Shh* (Seppala et al., 2014) as well as its targets *Nkx2.1* (Allen et al., 2007; Echevarria-Andino and Allen, 2020) and *Gli1* (Echevarria-Andino and Allen, 2020; Khonsari et al., 2013; Seppala et al., 2007) in this tissue in *Gas1* mutant mice.

Our findings now corroborate loss of SHH activity in the GAS1-deficient forebrain neuroepithelium *in vitro* and *in vivo*. Surprisingly, this defect may be attributed, at least in part, to a novel role for GAS1 in promoting NOTCH signaling, required to sustain the SHH activity domain in this forebrain organizer region. Loss of GAS1 impairs NOTCH-mediated facilitation of SHH signaling in neural progenitors and results in a failure to permanently establish the ventral SHH activity domain in the embryonic forebrain, which is the ultimate cause of forebrain and craniofacial anomalies in individuals lacking GAS1.

## RESULTS

### Reduced SHH and NOTCH pathway activities in the rostral ventral neuroepithelium of *Gas1* mutant mouse embryos

**Fig. 1.**
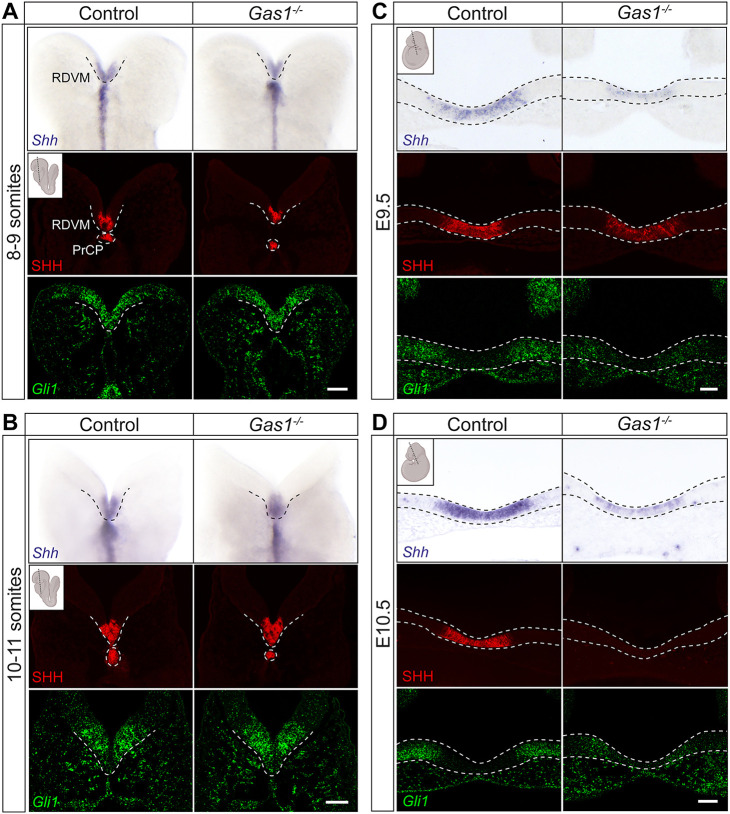
**GAS1 deficiency impedes persistence of the SHH activity domain in the murine embryonic forebrain.** SHH and *Gli1* expression patterns in control and *Gas1^−/−^* embryos at the indicated embryonic stages. Analyses were performed on whole-mount preparations (for *Shh* in A and B) or coronal sections thereof (as indicated in the respective insets produced using BioRender.com). (A,B) *Shh* transcript (blue; upper panels) and SHH protein (red; middle panels) are detectable in prechordal plate (PrCP) and rostral diencephalon ventral midline (RDVM), while *Gli1* transcripts are seen in the rostral ventral neuroepithelium (green; lower panels) at 8-9 somites (A) and 10-11 somites (B) in both genotypes. (C,D) Levels of *Shh* transcript (blue; upper panels) and SHH protein (red; middle panels), as well as *Gli1* transcript (green; bottom panels) are decreased at E9.5 (C) and completely lost at E10.5 (D) in the rostral ventral neuroepithelium of *Gas1*^−/−^ embryos compared with controls. Dotted lines indicate PrCP, RDVM or rostral ventral neuroepithelium, respectively. *n*=4 or 5 embryos per genotype and embryonic stage. Scale bars: 50 µm.

To dissect the contribution of GAS1 to SHH signaling during early neuroepithelial differentiation, we compared expression of *Shh* and its target *Gli1* (Lee et al., 1997) in *Gas1^−/−^* mouse embryos and their littermate controls. No differences were detected in *Shh* transcript and SHH protein in the PrCP and overlying RDVM, a major forebrain organizer region, at E8.5 (8-9 and 10-11 somites; Fig. 1A,B and Fig. S1A). By contrast, *Shh* and *Gli1* transcripts in the rostral ventral neuroepithelium of *Gas1^−/−^* embryos were decreased by E9.5 (Fig. 1C) and SHH protein was completely lost by E10.5, when compared with controls (Fig. 1D). These findings extended earlier observations of reduced expression of *Shh* (Seppala et al., 2014) at E12.5 or *Nkx2.1* (Allen et al., 2007; Echevarria-Andino and Allen, 2020) and *Gli1* (Echevarria-Andino and Allen, 2020; Khonsari et al., 2013; Seppala et al., 2007) at E9.5-10.5 in the ventral forebrain neuroepithelium of *Gas1* mutant mice. Importantly, our findings revealed that GAS1 is not essential for SHH signaling in this tissue. Rather, it promotes persistence of this SHH activity domain, initially established in the absence of this receptor.

At E8.5, expression of *Shh* in the PrCP provides the major source of the morphogen to pattern the overlying RDVM (Dale et al., 1997). To exclude a primary defect in this source as the reason for loss of SHH activity in *Gas1^−/−^* embryos at later embryonic stages, we quantified the size of the mutant PrCP based on *Shh* fluorescence *in situ* hybridization (FISH) on E8.5 coronal forebrain sections. These studies failed to detect any difference in the size of the *Shh* expression domain in the PrCP comparing control and *Gas1^−/−^* embryos (Fig. S1B,C).

**Fig. 2.**
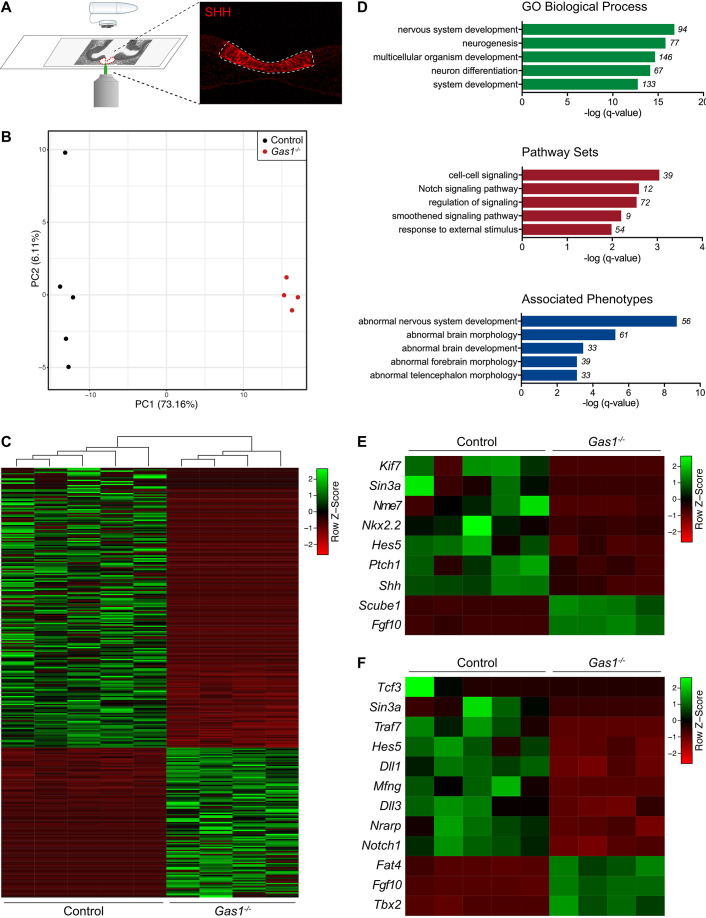
**Global transcriptomics indicate defects in NOTCH signaling in the GAS1-deficient rostral ventral forebrain.** (A) The SHH expression domain (as highlighted by immunodetection of SHH) in the rostral ventral midline of murine E10.0 forebrain sections was isolated by laser capture microdissection and subjected to bulk RNA-sequencing as detailed in the supplementary Materials and Methods. (B,C) Principal component analysis (PCA; B) and column-based hierarchical clustering heatmap (C) for all 324 identified differentially expressed genes (DEGs) of replicate pools (five embryos per pool) of control (*n*=5) and *Gas1*^−/−^ (*n*=4) tissue samples are shown. (D) Gene ontology (GO) term enrichment analysis, including the categories ‘biological process’, ‘enriched pathway sets’ and ‘associated phenotypes’. The five top hits for each category are shown in decreasing order of evidence based on GO term enrichment test q-value. Numbers indicate the quantity of DEGs related to the respective term. (E,F) Heatmap of DEGs associated with the GO terms ‘smoothened signaling pathway’ (E) and ‘NOTCH signaling pathway’ (F).

To elucidate the reasons for loss of SHH activity in *Gas1^−/−^* embryos at later stages of development, we performed comparative bulk RNAseq of the microdissected rostral ventral neuroepithelium from E10.0 *Gas1^+/+^* and *Gas1^−/−^* embryos (Fig. 2A). Global changes in the transcriptomes were determined by principal component analysis (Fig. 2B) and by hierarchical clustering of 324 identified differentially expressed genes (DEGs; Fig. 2C and Table S3). Gene ontology (GO) term enrichment analysis identified the expected changes in gene expression related to nervous system development, neurulation and Hedgehog signaling (Fig. 2D). Changes in DEGs included decreased levels of transcripts for *Shh*, *Ptch1* and *Nkx2.2* (Fig. 2E and Table S1). In addition, manual query of the RNAseq data for established downstream targets of the SHH signaling pathway identified statistically significant decreases in expression of *Isl1*, *Pomc1*, *Nkx2.1* and *Wnt5a* (Fig. S2), as well as a concordant upregulation of *Fgf10*, *Tbx2* (Fig. 2E,F) and *Tbx3* (Fig. S2). These changes recapitulated phenotypes seen in *Shh* mutant mice (Carreno et al., 2017; Corman et al., 2018; Crane-Smith et al., 2021; Szabo et al., 2009) and further substantiated a role for GAS1 in control of SHH activity in the developing forebrain neuroepithelium.

In addition to the anticipated change in SHH target gene expression, global transcriptomics identified unexpected alterations in NOTCH signaling in the ventral forebrain midline of *Gas1* mutants (Fig. 2D). NOTCH pathway components have been shown to be expressed in the rostral ventral neuroepithelium of the mouse embryo from E8.5 onwards (Ware et al., 2014), but no function for GAS1 in modulating NOTCH signaling in this domain had been described so far. In detail, our RNAseq data revealed decreased transcript levels for the DEGs NOTCH receptor 1 (*Notch1*), Delta-like proteins 1 (*Dll1*) and 3 (*Dll3*), Hes family bHLH transcription factor 5 (*Hes5*), NOTCH-regulated ankyrin repeat protein (*Nrarp*), and maniac fringe (*Mfng*) (Fig. 2F and Table S2). Alterations in NOTCH activity in *Gas1^−/−^* embryos were further supported by expression analyses of genes affected by NOTCH deficiency in other models (Ratié et al., 2013; Ware et al., 2016). Genes also downregulated in the rostral forebrain neuroepithelium of *Gas1* mutant mice included the transcription factor *Hey1*, which is a NOTCH target in the murine forebrain neuroepithelium (Ware et al., 2014) (Fig. S3). Of note, some targets, upregulated upon NOTCH pathway disruption in mouse and chick models (Ratié et al., 2013; Ware et al., 2016), were downregulated in the *Gas1^−/−^* ventral midline, including *Ascl1*, *Stmn2* and *Slit1* (Fig. S3).

**Fig. 3.**
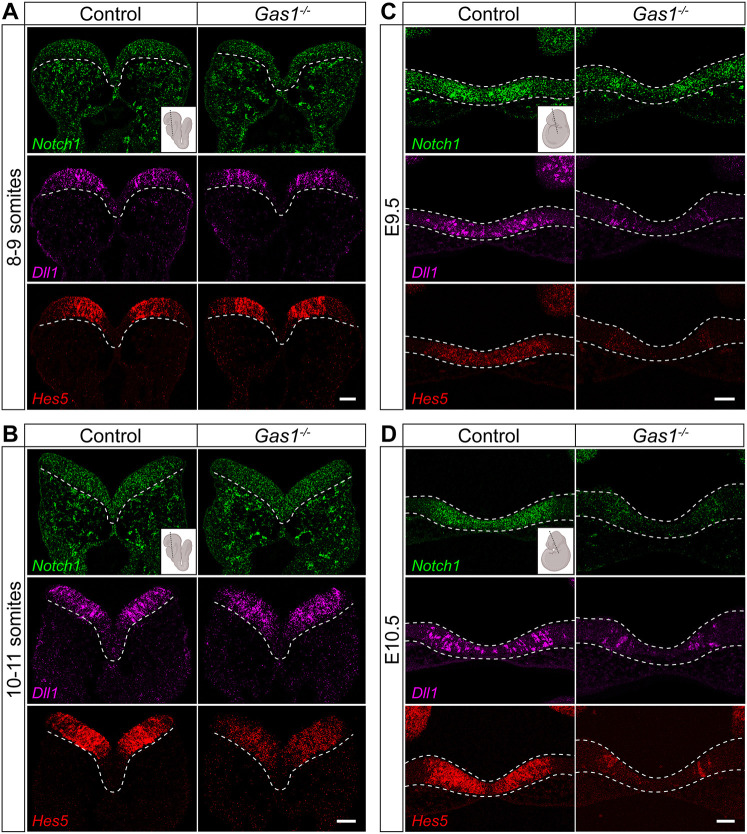
**GAS1 deficiency disrupts NOTCH signaling in the murine embryonic forebrain.** Detection of *Notch1*, *Dll1* and *Hes5* transcripts on coronal sections of control and *Gas1^−/−^* embryos at the indicated embryonic stages (plane of section indicated in the insets produced using BioRender.com). (A,B) While *Notch1* (green; upper panels) and *Dll1* (purple; middle panels) transcript levels in the rostral neuroepithelium are comparable between genotypes, transcript levels for *Hes5* (red; bottom panels) are normal at 8-9 somites (A) but decreased at 10-11 somites (B) in *Gas1*^−/−^ embryos compared with controls. *n*=4-6 embryos per genotype and somite stage (C,D) Transcript levels for *Notch1* (green; upper panels), *Dll1* (purple; middle panels) and *Hes5* (red; bottom panels) are reduced in the rostral ventral diencephalon of E9.5 (C) and E10.5 (D) *Gas1*^−/−^ embryos when compared with controls. *n*=8 embryos per genotype and embryonic stage. Scale bars: 50 µm. Dotted lines indicate rostral ventral neuroepithelium.

Impaired NOTCH pathway activity was further validated by expression analyses in *Gas1^−/−^* embryos. In detail, *Hes5* expression in the rostral neuroepithelium was reduced as early as E8.5 (10-11 somites; Fig. 3B), a time point coinciding with *Gas1* expression in this tissue (Fig. S4A). Transcripts for *Hes5*, and also for *Notch1* and *Dll1* in the rostral ventral neuroepithelium of mutants, were further reduced at E9.5 (Fig. 3C) and E10.5 (Fig. 3D). Notably, a reduction in *Hes5* expression at E8.5 preceded defects seen in the SHH pathway in *Gas1* mutants at E9.5 (Fig. 1C); and they were specific to the forebrain neuroepithelium as no changes in expression of *Notch1*, *Dll1* or *Hes5* were detected in the spinal cord of mutant when compared with control embryos (Fig. S4B).

### GAS1 promotes activation of NOTCH1 to facilitate SHH-dependent ventral neuroepithelial cell fate specification

To further dissect the molecular mechanism underlying GAS1 function in the rostral neuroepithelium, we established isogenic human induced pluripotent stem cell (iPSC) lines, either wild type or genetically deficient for *GAS1* (*GAS1* KO; Fig. S5A,B). Loss of *GAS1* did not impact pluripotency of iPSCs, as shown by normal expression of pluripotency markers (Fig. S5C-F) and by their ability to generate all three germ layers (Fig. S5G-J).

**Fig. 4.**
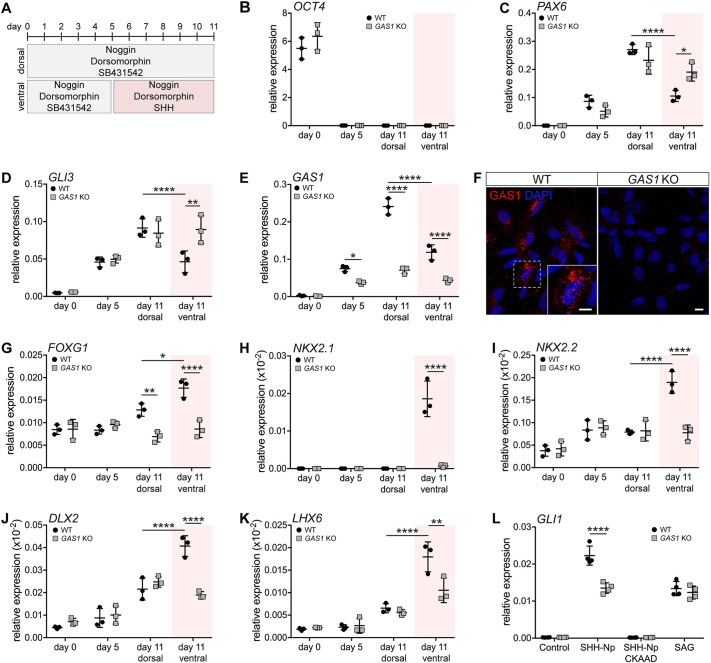
**Failure to induce SHH-dependent ventral neuroepithelial cell fate in GAS1-deficient human iPSCs.** (A) Protocol for neuroectodermal differentiation of human iPSCs to neural progenitor cells (NPCs). Cells received either SB431542 for 11 days (dorsal fate) or for only 5 days, followed by treatment with SHH for 6 days (ventral fate). (B-E) Quantitative (q) RT-PCR analysis of transcript levels for *OCT4* (B), *PAX6* (C), *GLI3* (D) and *GAS1* (E) at the indicated time points of dorsal or ventral neuroectodermal differentiation. *n*=3 biological replicates per genotype. (F) Immunodetection of GAS1 (red) in wild-type but not *GAS1* KO NPCs. Inset depicts a higher magnification of the boxed region in the overview image. Scale bars: 10 µm. (G-K) qRT-PCR analysis of transcript levels for *FOXG1* (G), *NKX2.1* (H), *NKX2.2* (I), *DLX2* (J) and *LHX6* (K) at the indicated time points of dorsal or ventral neuroectodermal differentiation. *n*=3 biological replicates per genotype. (L) Relative transcript levels of *GLI1*, as determined by qRT-PCR in NPCs at day 8-10 of neuroectodermal differentiation. Cells had been treated overnight with control medium or with medium containing 200 nM smoothened agonist (SAG) or SHH-Np, in the absence or presence of 50 nM cyclopamine-KAAD (CKAAD). *n*=4 biological replicates per genotype and condition. Levels in B-E and G-L are given as CT values normalized to transcript levels of *GAPDH* (2^−ΔCT^±s.d.). Statistical analyses were performed by two-way ANOVA with a Bonferroni post-hoc test. *P<0.05, **P<0.01, ****P<0.0001.

Next, wild-type and *GAS1* KO iPSCs were subjected to differentiation into neural progenitor cells (NPCs) of dorsal or ventral cell identity using established protocols (Fig. 4A) (Chambers et al., 2009; Flemming et al., 2020). When treated with noggin, dorsomorphin and small molecule SB431542 to block BMP and TGFβ signaling, both wild-type and *GAS1* KO iPSCs downregulated the pluripotency marker OCT4 and induced the neuroectodermal marker PAX6 (Fig. 4B,C; Fig. S6A,B). Consistent with adopting a dorsal neural progenitor fate, both genotypes induced *GLI3*, an inhibitor of the SHH pathway (Fig. 4D). In addition, wild-type, but not KO, cells induced expression of *GAS1* (Fig. 4E,F), a negative SHH transcription target (Allen et al., 2007). By contrast, when iPSCs were treated with SHH, instead of SB431542, to induce a ventral neural progenitor fate (schematic in Fig. 4A), *GAS1* KO cells failed to efficiently repress *PAX6* and *GLI3* (Fig. 4C,D), or to induce the ventral markers *FOXG1*, *NKX2.1*, *NKX2.2*, *DLX2* and *LHX6* (Fig. 4G-K). Such a cellular response to SHH was readily seen in wild-type cells adopting a ventral cell fate (day 11, ventral; Fig. 4C-K). Loss of SHH-dependent repression of PAX6 or induction of NKX2.1 in mutant NPCs was confirmed by immunocytochemistry (Fig. S6B,C). These data documented the inability of GAS1-deficient NPCs to adopt a ventral cell fate, likely due to their impaired response to ventralizing signals provided by SHH.

To substantiate the inability of *GAS1* KO cells to respond to SHH, we tested *GLI1* transcript levels in NPCs treated with conditioned medium from HEK293 cells secreting SHH-Np (Christ et al., 2012). SHH-Np induced *GLI1* transcription in wild-type NPCs to a much greater extent than in *GAS1* KO NPCs (SHH-Np; Fig. 4L), a response blocked by the hedgehog inhibitor cyclopamine-KAAD in both cell types (SHH-Np+CKAAD; Fig. 4L). Treatment with a smoothened (SMO) agonist (SAG) resulted in a similar induction of *GLI1* in both genotypes, indicating pathway integrity in *GAS1* KO cells downstream of SMO (SAG; Fig. 4L). Taken together, our findings substantiated iPSC-derived NPCs as a faithful model for studying neural progenitor fate decisions, and the importance of GAS1 for interpreting ventralizing signals provided by SHH to this cell type.

**Fig. 5.**
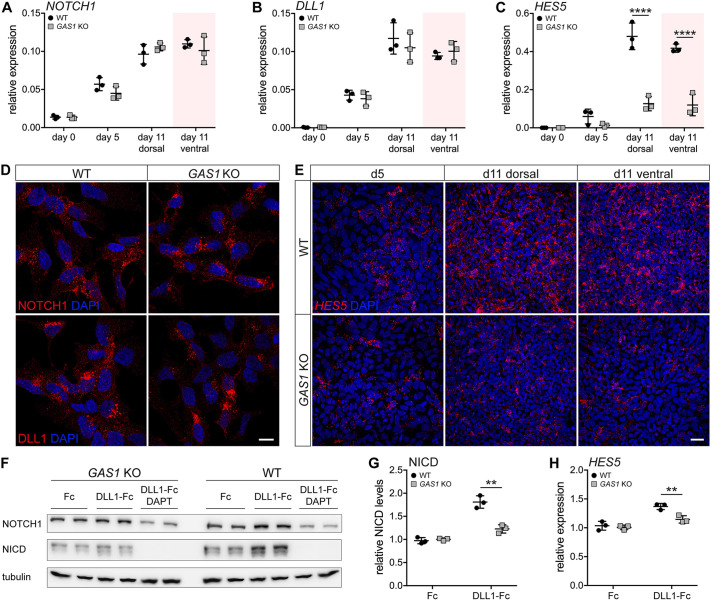
**GAS1 promotes NOTCH1 signaling in NPCs.** (A-C) Quantitative (q) RT-PCR analysis of relative transcript levels for *NOTCH1* (A), *DLL1* (B) and *HES5* (C) in wild-type and *GAS1* KO iPSCs at the indicated time points of dorsal or ventral neuroectodermal differentiation. Levels are depicted as CT values normalized to transcript levels of *GAPDH* (2^−ΔCT^±s.d.). *n*=3 biological replicates per genotype and time point. Statistical differences were analyzed by two-way ANOVA with a Bonferroni post-hoc test (****P<0.0001). (D) Immunodetection of NOTCH1 (red; upper panels) and DLL1 (red; lower panels) in wild-type and *GAS1* KO NPCs. Nuclei were counterstained with DAPI. Scale bar: 10 µm. (E) Detection of *HES5* transcripts in wild-type and *GAS1* KO NPCs at the indicated time points of dorsal or ventral neuroectodermal differentiation. Nuclei were counterstained with DAPI. Scale bar: 25 µm. (F) Western blot analysis of levels of full-length NOTCH1 and the NOTCH1 intracellular domain (NICD) in total lysates of wild-type and *GAS1* KO NPCs. Beforehand, cells were treated overnight with control Fc (Fc) or with recombinant DLL1-Fc-conjugated (DLL1-Fc) magnetic beads in the presence or absence of 25 µM γ-secretase inhibitor DAPT. Detection of tubulin served as loading control. (G) NICD levels in wild-type and *GAS1* KO NPCs treated with control Fc or with DLL1-Fc were determined by densitometric scanning of replicate western blots as exemplified in F. Levels are given as relative to the control condition (set to 1±s.d.). *n*=3 experiments with two or three replicates/experiment. (H) *HES5* transcript levels in wild-type and *GAS1* KO NPCs treated overnight with control Fc or DLL1-Fc-conjugated magnetic beads were determined by qRT-PCR. Levels are given as ΔCT values normalized to Fc-treated cells (2^−ΔΔCT^±s.d.). *n*=3 experiments. Statistical significances in G and H were determined using an unpaired, two-tailed *t*-test. **P<0.01.

In line with our gene expression data from *Gas1* mutant embryos, *GAS1* KO NPCs also failed to activate the NOTCH pathway during neuroectodermal differentiation. Thus, despite normal expression of *NOTCH1* and *DLL1* transcripts and proteins (Fig. 5A,B,D), induction of *HES5* transcript and protein levels was much lower in *GAS1* KO when compared with wild-type NPCs (Fig. 5C,E). This defect was seen for dorsal and ventral cell fates alike, documenting a SHH-independent role for GAS1 in NOTCH signaling. GAS1 deficiency impacted *Notch1* and *Dll1* expression in E9.5-10.5 embryos (Fig. 3C,D) but not in NPCs (Fig. 5A,B). This distinction likely reflected the fact that iPSC-derived NPCs recapitulate an early stage of neuroepithelial differentiation. Importantly, enhancing or abrogating SHH activity by SHH-Np and SAG or by cyclopamine, respectively, did not impact *HES5* transcript levels in wild-type NPCs (Fig. S7). These findings confirmed that SHH does not control expression of NOTCH pathway components in neural progenitors and that loss of SHH activity was not the primary cause of NOTCH pathway deficiency in *GAS1* KO NPCs.

The impact of GAS1 on NOTCH signaling manifested at the level of ligand-induced processing of the receptor polypeptide, as shown by quantification of the NOTCH1 intracellular domain (NICD) produced in response to treatment with its ligand DLL1. In wild-type NPCs, treatment with recombinant DLL1 increased NICD levels when compared with the control condition (Fig. 5F,G). NICD formation in wild-type NPCs was blocked by addition of the γ-secretase inhibitor DAPT (Fig. 5F,G). DLL1-induced NICD production correlated with increased levels of *HES5* transcripts in wild-type cells, faithfully recapitulating established NICD actions in this cell type (Fig. 5H). By contrast, DLL1-induced NICD generation and downstream signal transduction was completely lost in *GAS1* KO NPCs as the levels of NICD (Fig. 5F,G) as well as *HES5* transcripts (Fig. 5H) did not increase above the levels seen in cells in the absence of the ligand. These findings documented a complete loss of ligand-induced NOTCH activity in NPCs lacking GAS1.

**Fig. 6.**
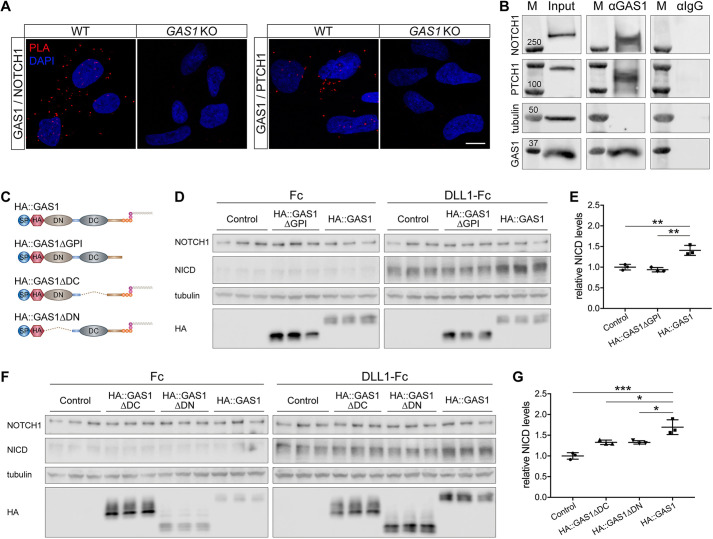
**GAS1 interacts with NOTCH1 to enhance NICD processing.** (A) Proximity ligation assay documenting proximity of GAS1 and NOTCH1 (red signal; left panels) as well as GAS1 and PTCH1 (red signal, right panels) in wild-type NPCs. No PLA signal was detected in *GAS1* KO cells. (B) Co-immunoprecipitation experiments using wild-type NPCs. The presence of GAS1, PTCH1 and NOTCH1 in total cell lysates is shown in the Input lane. In the αGAS1 lane, co-immunoprecipitation of NOTCH1 and PTCH1 using anti-GAS1 antiserum is documented. Detection of tubulin served as loading control (in Input lane) and as negative control for specificity of the antiserum (in αGAS1 lane). No proteins were immunoprecipitated using control anti-IgG antiserum (αIgG lane). The molecular weights of protein standards (kDa) are shown in lane M. (C) HA-tagged expression constructs for full-length human GAS1 (HA::GAS1) or truncated variants lacking the GPI anchor (HA::GAS1ΔGPI), or DC (HA::GAS1ΔDC) or DN (HA::GAS1ΔDN) domains. Constructs were transiently transfected into HEK293 cells to analyze their effect on NOTCH1 activation. (D,E) Parental HEK293 cells (Control) or cells expressing HA::GAS1 or HA::GAS1ΔGPI were treated with Fc or recombinant DLL1-Fc-conjugated magnetic beads. Levels of NICD were quantified 16 h later by western blot analysis (D) and densitometric scanning of replicate blots (E). Detection of tubulin and NOTCH1 served as loading controls, detection of HA as a GAS1 expression control. Data in E are mean±s.d. of NICD levels (normalized to tubulin) relative to controls treated with DLL1-Fc (set at 1). *n*=3 experiments with two or three replicates/experiment. DLL1-induced production of NICD is higher in HA::GAS-expressing when compared with HA::GAS1ΔGPI-expressing or control cells. (F,G) Experiments as in D and E but using control or HEK293 cells expressing HA::GAS1, HA::GAS1ΔDC or HA::GAS1ΔDN. *n*=3 experiments with two or three replicates/experiment. DLL1-induced production of NICD is higher in HA::GAS1 when compared with control cells or cells expressing HA::GAS1ΔDC or HA::GAS1ΔDN. Statistical differences in E and G were determined by one-way ANOVA with a Bonferroni post-hoc test. *P<0.05, **P<0.01, ***P<0.001.

To investigate the molecular mechanism of GAS1 action in NOTCH signaling, we performed proximity ligation assays, demonstrating the close proximity of GAS1 and NOTCH1 in wild-type NPCs (Fig. 6A). Immunoprecipitation (IP) assays further showed that GAS1 co-immunoprecipitates with PTCH1 (a known interaction) but also with NOTCH1 (Fig. 6B), further arguing that GAS1 and NOTCH1 physically interact. To further dissect the domain requirements for GAS1 in NOTCH1 activation, we transfected HEK293 cells with hemagglutinin (HA)-tagged full-length or truncated variants of GAS1 (Fig. 6C) and determined endogenous NICD production in response to DLL1 application. Whereas full-length GAS1 induced DLL1-dependent NICD production, GAS1 lacking the GPI anchor did not (Fig. 6D,E). In addition, deletion of the extracellular DN or DC domains in GAS1 eliminated the ability of the receptor to induce NICD production (Fig. 6F,G). These findings documented that full-length membrane-tethered GAS1 is required to promote NOTCH1 signaling in neural progenitors.

### Ectopic NOTCH signaling rescues loss of SHH activity in the GAS1-deficient forebrain neuroepithelium

**Fig. 7.**
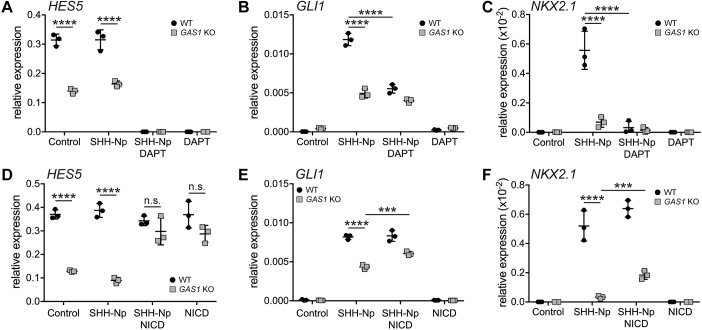
**GAS1 enhances SHH activity in iPSC-derived NPCs by facilitating NOTCH signaling.** (A-C) Relative transcript levels of *HES5* (A), *GLI1* (B) and *NKX2.1* (C) were determined by qRT-PCR in wild-type and *GAS1* KO NPCs at day 10 of neuroectodermal differentiation. Cells were treated either with control or SHH-Np-conditioned medium (in the absence or presence of DAPT) or with DAPT alone for 3 days. *n*=3 biological replicates per genotype and condition. (D-F) Relative transcript levels of *HES5* (D), *GLI1* (E) and *NKX2.1* (F) in wild-type or *GAS1* KO NPCs at day 8 of neuroectodermal differentiation. Cells were treated with control or SHH-Np-conditioned medium overnight in the absence or presence of lentiviral-induced NICD overexpression. *n*=3 biological replicates per genotype and condition. Levels are depicted as CT values normalized to transcript levels of *GAPDH* (2^−ΔCT^±s.d.). Statistical analyses were performed by two-way ANOVA with a Bonferroni post-hoc test. ***P<0.001, ****P<0.0001.

NOTCH has been shown to facilitate SHH signaling in the retina and spinal cord of mouse, chick and zebrafish models (Huang et al., 2012; Jacobs and Huang, 2019; Kong et al., 2015; Ringuette et al., 2016; Stasiulewicz et al., 2015). To investigate a similar role for NOTCH in the rostral neuroepithelium, and the relevance of GAS1 in this process, we studied the interdependency of SHH and NOTCH pathways in NPCs. Levels of *HES5* were always lower in *GAS1* KO when compared with wild-type NPCs, irrespective of the presence or absence of SHH-Np (Fig. 7A), consistent with the notion that GAS1 activation of NOTCH is SHH independent. By contrast, induction of *GLI1* and *NKX2.1* expression in wild-type NPCs by addition of SHH-Np was reduced (*GLI1*) or even completely lost (*NKX2.*1) by blockade of NOTCH signaling using DAPT (Fig. 7B,C). In fact, *GLI1* and *NKX2.1* transcript levels in wild-type cells treated with SHH-Np and DAPT were comparable with levels in *GAS1* KO cells treated with SHH-Np in the absence of DAPT. These findings further argued that GAS1-dependent activation of NOTCH1 is a major contributor to SHH signal strength in neural progenitors.

To further substantiate the relevance of GAS1-dependent activation of NOTCH1 for SHH signaling, we tested SHH activity in *GAS1* mutant cells following ectopic induction of NOTCH1 activity. In these experiments, lentiviral overexpression of NICD rescued the defect in *HES5* induction in *GAS1* KO NPCs independently of SHH as levels of this transcript were comparable in NICD-treated wild-type and KO NPCs, both in the presence or absence of SHH-Np (Fig. 7D). Importantly, NICD overexpression partially rescued SHH-dependent *GLI1* and *NKX2.1* induction in *GAS1* KO NPCs treated with SHH-Np and NICD when compared with treatment with SHH-Np only (Fig. 7E,F).

**Fig. 8.**
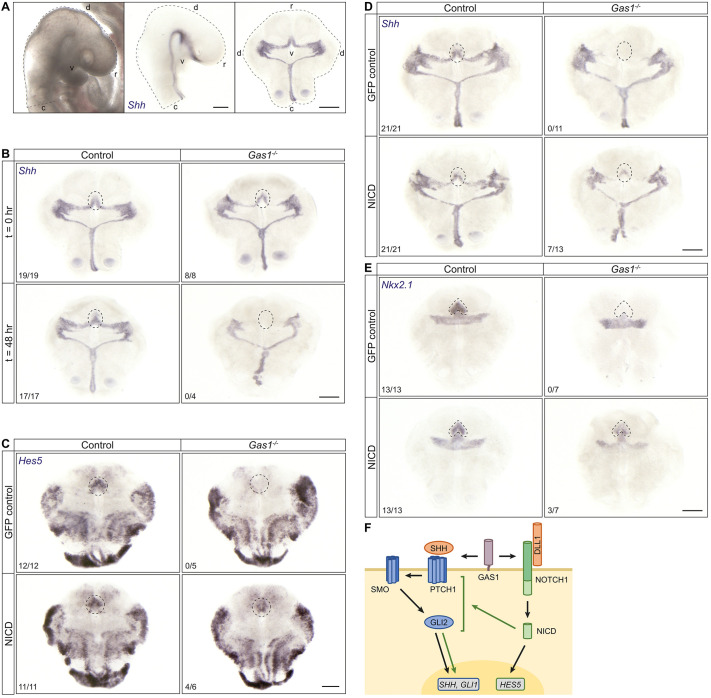
**Ectopic expression of NICD rescues loss of the SHH activity in GAS1-deficient rostral ventral forebrain explants.** (A) Preparation of cephalic explants as detailed elsewhere (Christ et al., 2012; Echevarria et al., 2001). Scale bars: 500 μm. (B) Cephalic explants of E9.5 control and *Gas1^−/−^* embryos were fixed 2 h (t=0 h) or 48 h (t=48 h) after dissection and subjected to *in situ* hybridization (ISH) for *Shh*. The expression domain for *Shh* in the rostral ventral neuroepithelium (marked by dotted circles) is seen in *Gas1^−/−^* embryos at t=0 h, albeit slightly reduced when compared with wild type. However, this expression domain is completely lost in *Gas1^−/−^* embryos at t=48 h. (C-E) Cephalic explants of E9.5 control or *Gas1^−/−^* embryos were treated with lentiviral constructs encoding EF.PGK.GFP (GFP control) or NICD-pcw107-V5 (NICD), and subjected to ISH for *Hes5* (C), *Shh* (D) or *Nkx2.1* (E) 48 h later. Expression domains for *Hes5*, *Shh* and *Nkx2.1* in the rostral ventral neuroepithelium (circled by dotted lines) are absent from EF.PGK.GFP-treated *Gas1^−/−^* when compared with wild-type explants, but are partially rescued in *Gas1^−/−^* explants by NICD-pcw107-V5. The number of explants with robust signal for *Hes5*, *Shh* or *Nkx2.1* in the rostral ventral neuroepithelium, out of all explants analyzed, are given for each condition and genotype. Scale bars: 500 µm. (F) Model for GAS1 integrating SHH and NOTCH signaling pathways in neural progenitor cells.

So far, our findings corroborated a role for GAS1 in NOTCH-dependent facilitation of SHH signaling in cultured NPCs. To confirm the relevance of this activity for SHH action in the developing forebrain, we tested the ability of NOTCH signaling to rescue loss of SHH activity in the *Gas1^−/−^* rostral ventral neuroepithelium. To do so, we used cephalic explants, a utilitarian model to study neuroepithelial differentiation *ex vivo* (Christ et al., 2012; Echevarria et al., 2001). In the protocol used here, cephalic explants were isolated from E9.5 wild-type and *Gas1^−/−^* embryos, and cultured for 48 h, followed by gene expression analyses using ISH (Fig. 8A). In *Gas1* mutant explants, correct, albeit slightly reduced, expression of *Shh* was seen at E9.5 in the rostral ventral neuroepithelium (t=0 h; Fig. 8B). This expression domain was completely lost after 2 days in culture (t=48 h; Fig. 8B). By contrast, *Shh* expression sustained in the rostral ventral neuroepithelium of wild-type explants (Fig. 8B). These findings recapitulated our *in vivo* data that GAS1 was required to sustain the *Shh* expression domain, initially established normally around E8.5 in the *Gas1* mutant forebrain. Thus, cephalic explants represented a faithful model to recapitulate *Shh* defects observed in the GAS1-deficient rostral neuroepithelium *in vivo*.

Next, we treated cephalic explants with lentiviral constructs encoding NICD to ectopically induce NOTCH signaling. In line with this strategy, expression of *Hes5*, which is absent from GAS1-deficient explants treated with control virus, was rescued in mutants by ectopic expression of NICD (Fig. 8C). Importantly, overexpression of NICD also increased *Shh* transcript levels in the rostral ventral neuroepithelium in approximately half of the *Gas1^−/−^* explants to levels similar to that in wild-type tissue (Fig. 8D). Similarly, NICD expression also partially rescued levels of *Nkx2.1* in the rostral ventral neuroepithelium of mutant explants (Fig. 8E). As a negative control, no rescue of *Shh* or *Nkx2.1* expression was detected in *Gas1^−/−^* explants treated with a control virus (Fig. 8D,E).

In conclusion, our findings identified a novel role for GAS1 in integrating SHH and NOTCH signaling pathways, a function specific to neural progenitors in the rostral forebrain neuroepithelium destined to adopt a ventral cell fate (Fig. 8F). According to our model, GAS1 interacts with PTCH1 to promote SHH-dependent gene expression, including induction of *Shh*, *Nkx2.1* or *Gli1*. However, GAS1 also interacts with NOTCH1 to facilitate ligand-induced production of NICD. GAS1-dependent NICD production induces prototypic NOTCH targets, such as *Hes5* and *Hey1*, but it also acts on the SHH pathway to increase strength and persistence of SHH signal reception. The latter mode of action is unclear at present but, based on work by others, may act at the level of several HH pathway components, including PTCH1, SMO or GLI2.

## DISCUSSION

Previous studies have identified multiple developmental abnormalities in mouse models and patients carrying mutations in *GAS1*. These defects have been phenotypically characterized in great detail in *Gas1^−/−^* mice and include microforms of HPE, but also a range of craniofacial anomalies, such as midfacial hypoplasia, premaxillary incisor fusion, cleft palate or malformation of the anterior pituitary (Allen et al., 2007; Echevarria-Andino and Allen, 2020; Khonsari et al., 2013; Martinelli and Fan, 2007; Seppala et al., 2007, 2014). Malformations phenocopy aspects of SHH deficiency, including hypothalamic defects and pituitary hypoplasia (Carreno et al., 2017; Zhao et al., 2012). Phenotypes increase in severity with haploinsufficiency for *Shh* (Allen et al., 2007; Khonsari et al., 2013; Martinelli and Fan, 2007; Seppala et al., 2007), documenting interaction of *Gas1* and *Shh* in formation of the midline and structures derived thereof. Increasing or decreasing GAS1 activity in the neural tube of chick or mouse positively correlates with SHH pathway activity, corroborating an agonistic role for GAS1 in the graded response of neuroepithelial cells to ventralizing signals by this morphogen (Allen et al., 2007; Khonsari et al., 2013; Martinelli and Fan, 2007; Seppala et al., 2007). Although studies on the cellular response to SHH mainly concern a role for GAS1 in patterning of the caudal neural tube and limbs (Allen et al., 2011, 2007; Martinelli and Fan, 2007), they also serve as an explanatory model for GAS1 action in SHH signaling in the rostral neuroepithelium. This assumption is supported by loss of expression of *Shh* and downstream targets in this tissue around E9.5-12.5 (Allen et al., 2007; Echevarria-Andino and Allen, 2020; Khonsari et al., 2013; Seppala et al., 2014).

Our studies aimed at corroborating a role for GAS1 in the cellular response of neural progenitors of the forebrain neuroepithelium to SHH signals. To do so, we applied unbiased as well as targeted approaches of molecular phenotyping of the forebrain neuroepithelium during early neurulation (E8.5-10.5), and we queried our findings by modeling morphogen actions in iPSC-derived NPCs. NPCs faithfully recapitulate the *in vivo* response of neuroepithelial cells to morphogen signals and the consequential dorsal versus ventral cell fate choices (Fig. 4B-K). In addition, this cell model faithfully recapitulates defects in the immediate cellular response to SHH signals observed in the GAS1-deficient forebrain neuroepithelium *in vivo* and *ex vivo*. Importantly, this cell model enables quantitative assessment of the cellular response to morphogen signals using agonists and antagonists, an experimental strategy difficult to apply to forebrain patterning *in vivo*.

Concerning the presumed role for GAS1 in the cellular response of the forebrain neuroepithelium to SHH, our studies extended previous work by confirming loss of morphogen expression and activity around E9.5 (Fig. 1C). These defects are consistent with SHH deficiency at early neurulation, as seen in *Shh* mutant mouse embryos (Carreno et al., 2017; Corman et al., 2018; Crane-Smith et al., 2021; Szabo et al., 2009). However, contrary to prior analysis that focused on developmental stages after E9.5, our data uncovered that GAS1 is not required for initial establishment of the SHH domain in the RDVM at E8.5, but is necessary to sustain its activity at later stages of neurulation. The same effect is also seen in cephalic explants (Fig. 8B). This mode of action distinguishes GAS1 from other SHH-binding proteins, such as LRP2, which is required for initial establishment of the SHH activity domain in the RDVM at E8.5 (Christ et al., 2012). A facilitatory role for GAS1 in SHH signal reception in neural progenitors is substantiated by quantitative assessment of their response to pathway stimulation. In these experiments, the response of *GAS1* KO cells to SHH-Np is lower than in wild-type cells, yet significant when compared with non-treated cells, and comparable with the extent of pathway stimulation seen with SAG in either genotype. Still, baseline stimulation of the SHH pathway in the absence of GAS1 is clearly insufficient to trigger a ventral cell fate decision (Fig. 4B-K), providing a molecular correlate for ventralizing defects seen in the *Gas1* mutant neural tube *in vivo* (Allen et al., 2007; Khonsari et al., 2013; Martinelli and Fan, 2007; Seppala et al., 2007).

Although a role for GAS1 in the response of the forebrain neuroepithelium to SHH may have been anticipated based on previous work, a role for this receptor in NOTCH signaling in this cell type is novel and surprising. In NPCs, loss of GAS1 completely abrogates the ability to induce the NOTCH target *HES5*, an effect independent of dorsal or ventral cell fate decisions (Fig. 5C,E). In addition, GAS1-deficient NPCs fail to respond to the NOTCH ligand DLL1 with induction of NICD production and *HES5* transcription (Fig. 5F-H). Deficiency in NOTCH signaling is confirmed in GAS1-deficient embryos as early as E8.5 (Fig. 3B). Phenotypes include features observed in mice with targeted disruption of the NOTCH pathway component RBPJ, such as loss of *Hes5* in the rostral ventral diencephalon (Ware et al., 2016). Some targets, such as *Ascl1*, which is upregulated upon NOTCH pathway disruption in the RBPJ KO mouse model (Ratié et al., 2013; Ware et al., 2016), were downregulated in the *Gas1^−/−^* ventral midline (Fig. S3). These distinctions are likely due to the fact that *Gas1^−/−^* mice still retain the activity of RBPJ, which acts as a transcriptional repressor in the absence of NICD (Castel et al., 2013). NOTCH signaling promotes progenitor cell maintenance in the developing CNS and controls neuronal/glial cell fate decisions (reviewed by Gaiano and Fishell, 2002). Specifically, recent work identified the importance of NOTCH signaling for maintenance and differentiation of prosencephalic structures, including hypothalamic neurons and pituitary gland. Consequently, NOTCH signaling defects in the RBPJ KO mouse model causes malformation of the pituitary gland (Aujla et al., 2015, 2013), a defect shared by GAS1-deficient mice (Khonsari et al., 2013). Although not explored in this study in detail, loss of NOTCH activity in the rostral neuroepithelium of *Gas1^−/−^* embryos may be expected to cause additional phenotypes related to NOTCH deficiency.

Concerning the molecular mechanism whereby GAS1 promotes NOTCH activation, this action likely involves direct interaction with NOTCH1, a co-receptor concept also operable for GAS1 action on PTCH1 (Izzi et al., 2011). At present, we can only speculate about the mode of GAS1 action in this context. Because the ability of GAS1 to promote NOTCH1 activation is lost when the GPI anchor is deleted, one may argue that GPI-anchored GAS1 targets NOTCH1 to specialized lipid raft compartments where secretases or ligands reside. Such a NOTCH-sorting function has been shown for GPI-anchored Cripto-1 (Watanabe et al., 2009). Whatever the mode of action, it is operable in the rostral but not the caudal neural tube, supporting a unique role for GAS1 in NOTCH signaling during early forebrain patterning.

Conceptually, GAS1 deficiency phenotypes may represent a combination of NOTCH and SHH defects, originating from independent functions of GAS1 in activation of PTCH1 and NOTCH1. More exciting is the hypothesis that both functions for GAS1 converge on its ability to promote SHH signal strength and persistence in the forebrain neuroepithelium (see schematic in Fig. 8F). NOTCH is known to exert some of its actions by facilitating SHH signal reception and maintaining SHH responsiveness in target cells. These actions may work through different mechanisms. On the one hand, NOTCH signaling has been shown to regulate the availability and stability of GLI proteins in mouse retinal progenitor cells (Ringuette et al., 2016) as well as in neural progenitor cells of the zebrafish spinal cord (Jacobs and Huang, 2019). On the other hand, NOTCH signals prime neural progenitor cells of the mouse and chick neural tube for response to SHH by regulating trafficking of PTCH1 and SMO (Huang et al., 2012; Kong et al., 2015; Stasiulewicz et al., 2015). Haploinsufficiency for NOTCH pathway components in some patients with HPE further suggests that NOTCH-dependent facilitation of SHH signaling is required for forebrain formation (Dupe et al., 2011). This conclusion is supported by loss of NOTCH-dependent SHH activity in the embryonic mouse and chick forebrains following pharmacological or genetic perturbation of NOTCH activity, defects that include disruption of the hypothalamo-pituitary axis (Hamdi-Rozé et al., 2020).

Although dissection of NOTCH-dependent versus NOTCH-independent effects of GAS1 on SHH signaling will be challenging *in vivo*, iPSC-based modelling of neural progenitor differentiation enables quantitative assessment of the contribution of both pathways to SHH signal strength. In wild-type NPCs, SHH-Np-induced gene expression is largely reduced (*GLI1*) or even completely abolished (*NKX2.1*) by blockade of NOTCH using DAPT (Fig. 7B,C). In support of a prominent role for NOTCH in SHH signal strength, SHH signaling defects in *GAS1* mutant NPCs can be partially rescued *in vitro* (Fig. 7E,F) and in cephalic explants *ex vivo* (Fig. 8D,E) by NICD. Although this experimental approach does not formally rule out a GAS1-independent role for NOTCH in SHH signal transduction, SHH-Np-induced expression of *GLI1* is comparable in wild-type and *GAS1* KO cells in the presence of DAPT, arguing that a major contribution to SHH signal strength in wild-type cells stems from the action of GAS1 on NOTCH (Fig. 7B).

In conclusion, our findings suggest a new concept concerning the role of SHH co-receptors in control of morphogen signaling in forebrain neuroepithelial cells. Specifically, they document that GAS1 acts as a co-receptor for both PTCH1 and NOTCH1 to integrate instructive signals by SHH and NOTCH ligands in this cell type; and they argue that loss of GAS1-dependent NOTCH activation may contribute to forebrain malformations in individuals carrying *GAS1* mutations.

## MATERIALS AND METHODS

### Mouse models

Mice carrying a targeted disruption of *Gas1* (Martinelli and Fan, 2007) have been described. The *Gas1* mutant line was kept by breeding *Gas1^+/−^* animals on a C57BL/6N genetic background. As no phenotypic differences in forebrain formation were observed between *Gas1^+/+^* and *Gas1^+/−^* embryos in this study, both genotypes were used as matched littermate controls for GAS1-deficient embryos. All animal experimentation was performed following approval by authorities of the State of Berlin (X9007/17). *In situ* hybridization (ISH) and immunohistology on mouse tissues were performed according to published protocols (Christ et al., 2012) and as detailed in the supplementary Materials and Methods.

### A GAS1-deficient human iPSC model

Human induced pluripotent stem cell line HPSI1113i-wetu_2 was kindly provided by the Wellcome Trust (Sanger Institute, UK) and used as wild-type control cell line (WT). iPSCs were cultured on Matrigel (354277, Corning) -coated culture plates in Essential 8 (E8) or E8 Flex medium (Gibco). Culture medium was changed daily. Cells were passaged every 3-4 days at a density of 70-80% using StemPro Accutase (Gibco) and 10 µM of Rock inhibitor Y27632 (SEL-S1049, Selleck Chemicals). A GAS1-deficient subclone of HPSI1113i-wetu_2 (*GAS1* KO) was generated by targeting the *GAS1* gene using the CRISPR/Cas9 system. Single guide RNA (sgRNA) was designed using the online software tool CHOPCHOP (Labun et al., 2019). sgRNA sequences were sense, CTCAACGACTGCGTGTGCGA; and antisense, TCGCACACGCAGTCGTTGAG. Annealed sgRNA oligonucleotides were cloned into the expression vector pSpCas9(BB)-2A-Puro V2.0 (PX459, Addgene plasmid #62988). Human iPSCs were transfected with the final sgRNA-plasmid construct using Lipofectamine3000 (Invitrogen) according to the manufacturer's instructions. Transfected cells were selected with 0.1 µg/ml puromycin for 1 week before seeding them at low density for single cell colony expansion. Clones were analyzed for successful deletion using the Phire Animal Tissue Direct PCR Kit (Thermo Fisher Scientific). Primer sequences used were CAAAGTCTTCAACGGGCTGC (forward) and CGGGCCATGTTCTCCTTGA (reverse). To confirm deletion of the targeted *GAS1* region, DNA sequencing was performed by LGC Genomics GmbH and data analyzed using the DNAStar SeqMan Software Version 13.0.0. Analysis of pluripotency by scorecard assay and differentiation in neuroepithelial progenitor cells is described in the supplementary Materials and Methods. All iPSC lines were regularly tested negative for mycoplasma.

### Microdissection and bulk RNAseq

Tissues were dissected from cryosections of the embryonic forebrain region by manually guided laser capture microdissection on a Zeiss Axio Observer Z1. Total RNA was isolated from the dissected tissue samples (RNA extraction kit, Qiagen) and used for cDNA library preparation (SMARTer Stranded Total RNA-Seq Pico Kit; Takara). Libraries were sequenced in a 2× 75 bp paired end run on an Illumina HiSeq 4000 system with 20 million reads per sample. For more details, see supplementary Materials and Methods.

### Generation of neural progenitor cells

Neural progenitor cells were differentiated from iPSCs using standard protocols based on inhibition of BMP and TGF signaling, as well as induction of SHH signaling (ventral fate). For more details, see supplementary Materials and Methods.

### Immunofluorescence staining and immunohistology of iPSCs/NPCs

Immunodetection of marker proteins was performed on monolayers of iPSCs or NPCs fixed with 4% paraformaldehyde and permeabilized with 0.5% Triton X-100. Proteins were visualized by overnight incubation with primary antibodies (at 4°C), followed by incubation with the respective secondary antibodies conjugated with Alexa Fluor fluorophores (1-2 h at room temperature). For more details, see supplementary Materials and Methods.

### Proximity ligation assay

Spatial proximity of target proteins was tested on fixed and permeabilized NPCs using primary antibodies directly conjugated with oligonucleotides (Duolink In Situ Probemaker), followed by rolling circle amplification using real-time PCR (Duolink In Situ Detection Reagent Kit Orange). Experiments were performed according to the manufacturer's instructions (Sigma-Aldrich). For more details, see supplementary Materials and Methods.

### Co-immunoprecipitation

Co-immunoprecipitation was performed in total protein lysates from NPCs using 10 μg/ml goat anti-GAS1 antibody (AF2636, R&D Systems) or a non-immune goat IgG control (01-6202, Invitrogen) and the Pierce Crosslink Magnetic IP/Co-IP Kit (Thermo Fisher Scientific), according to the manufacturer’s instructions. For more details, see supplementary Materials and Methods.

### Lentivirus production

For lentivirus production, HEK293TN cells were transfected with lentivirus envelope and packaging plasmids pMD2.D, pMDLg/pRRE and pRSV-Rev (Addgene plasmids #12259, #12251 and #12253, respectively), and the control plasmid EF.PGK.GFP (Addgene plasmid #17618) or the NICD-expressing plasmid NOTCH1 intracellular domain-pcw107-V5 (Addgene plasmid #64622). Cells were further cultivated for 2-3 days and virus particles purified from collected cell media overnight on ice using Lenti-X Concentrator (Clontech Laboratories). For more details, see supplementary Materials and Methods.

### SHH signaling in NPCs

NPCs of differentiation day 7-9 were incubated with 5-10% conditioned medium from control or SHH-Np-secreting HEK293 cells (Christ et al., 2012), 200 nM SAG (SML1314, Sigma-Aldrich), 50 nM cyclopamine-KAAD (239804, Calbiochem) or 25 µM DAPT (565770, Sigma-Aldrich) diluted in N2B27 medium overnight or for 3 days (for studies including DAPT). The medium was changed daily with freshly added compounds. For rescue experiments, NPCs from differentiation day 5 were dissociated using Accutase and seeded onto Matrigel-coated 24-well-plates at a density of 400,000 cells/well. Cells were transduced with 20 µl/well NICD or GFP control expressing lentivirus solutions and 8 µg/ml polybrene overnight. 48 h after transduction, cells were incubated overnight with 5-10% conditioned medium from control or SHH-Np secreting HEK293 cells. Cells were then subjected to gene expression analysis by quantitative real-time PCR (for more details, see the supplementary Materials and Methods).

### Analysis of NOTCH1 signaling in NPCs and HEK293

NPCs at differentiation day 7-9 were treated with recombinant DLL1-Fc (10184-DL, R&D Systems) or Fc control (110-HG, R&D Systems) coupled to Pierce Protein G Magnetic Beads (Thermo Fisher Scientific) and, where applicable, additionally with 25 µM DAPT overnight. Human GAS1 constructs (HA-hGAS1, HA-hGAS1ΔGPI, HA-hGAS1ΔDN and HA-hGAS1ΔDC) were generated by PCR-based cloning into the pCIG vector (Megason and McMahon, 2002) and confirmed by sanger sequencing. HEK293T cells were transfected with the various GAS1 constructs using Lipofectamine2000. 48 h after transfection, cells were incubated with DLL1-Fc or Fc control magnetic beads overnight. Cells were washed with PBS to remove beads and subjected to protein analysis by western blotting. For western blotting, cell lysates were subjected to standard SDS-polyacrylamide gel electrophoresis, followed by transfer onto nitrocellulose membranes. Bound proteins were detected using primary and horseradish peroxidase-conjugated secondary antibodies and the SuperSignal West Femto Maximum Sensitivity Substrate (Thermo Fisher Scientific) (for more details, see supplementary Materials and Methods). For each reaction, 20 µl protein G magnetic beads were first equilibrated in PBS and then incubated with 500 ng DLL1-Fc or Fc control overnight at 4°C on a rotator. The next day, the beads were washed and stored in PBS at 4°C until use.

### Rescue of SHH signaling in explants

Cephalic explants were prepared as described elsewhere (Christ et al., 2012; Echevarria et al., 2001) with minor modifications. Briefly, E9.5 embryos were dissected in DMEM without phenol red supplemented with 1×GlutaMAX and 1×penicillin-streptomycin (Gibco). Embryonic heads were opened along the dorsal midline and the floor plate was cut at the level of the cephalic flexure. Explants were placed on polycarbonate membrane filters of 0.8-1 µm pore size (Millipore) with the ventricular side facing up and transferred to 24-well-plates containing DMEM supplemented with 10% FCS, 1×GlutaMAX and 1×penicillin-streptomycin (Gibco). Explants were recovered for 2-3 h at 37°C with 5% CO_2_ and 95% humidity. 20 µl lentivirus solution containing NICD or GFP-encoding lentivirus particles was applied on top and explants were further cultivated for 48 h. After gently washing with culture medium and subsequently with PBS, the explants were fixed in 4% PFA at 4°C overnight before subjecting to whole-mount ISH for *Shh*, *Nkx2.1* or *Hes5*.

### Statistical analysis

Data are represented as mean±s.d. All statistical analyses were performed using GraphPad Prism 7.0. The applied statistical tests are indicated in the respective figure legends.

## Supplementary Material

Supplementary information

Reviewer comments
