# Supplementary material for: GAS1 is required for NOTCH-dependent facilitation of SHH signaling in the ventral forebrain neuroepithelium
Source: Development. 2021 Nov 11;148(21):dev200080. doi: 10.1242/dev.200080 (PMC8627604; doi:10.1242/dev.200080)
Supplement: Supplementary information [file develop-148-200080-s1.pdf]

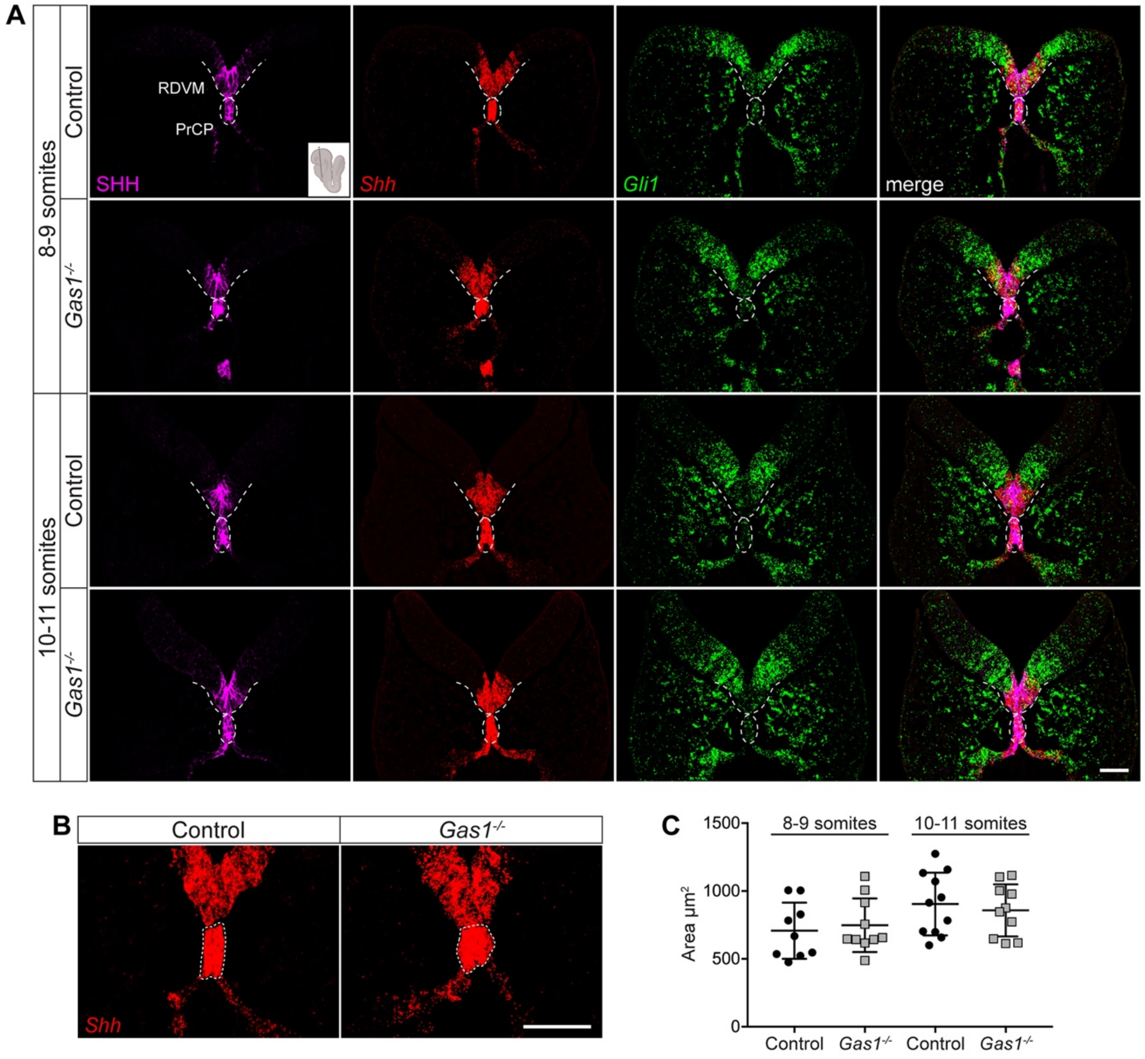

A
8-9 somites
10-11 somites
Control
Gas1-/-
RDVM
PrCP
SHH
Shh
Gli1
merge
B
Control
Gas1-/-
Shh
C
8-9 somites
10-11 somites
Area µm2
1500
1000
500
0
Control
Gas1-/-
Control
Gas1-/-

Fig. S1. Expression and activity of SHH are not impacted in Gas1-/- embryos during early neurulation.

(A) SHH protein (magenta; left panels) as well as Shh (red; middle left panels) and Gli1 transcripts (green; middle right panels) were co-detected on coronal sections from E8.5 control or Gas1-/- embryos (8-9 and 10-11 somite stages) using immunohistology or fluorescence in situ hybridization (FISH), respectively. Both single and merged channel (right panels) configurations are shown for each section. Dotted lines demarcate prechordal plate (PrCP) and rostral diencephalon ventral midline (RDVM). The inset (adapted from BioRender.com) illustrates the plane of section for all panels. n=3 embryos per somite stage and genotype. (**B**) Detection of *Shh* transcripts on coronal sections of E8.5 control and *Gas1*$^{-/-}$ embryos using FISH. Image magnified from panel (A). Dotted lines demarcate the PrCP. (**C**) The area of the *Shh* expression domain in the PrCP of control and *Gas1*$^{-/-}$ embryos at 8-9 and 10-11 somites was quantified using FISH (as exemplified in B). Areas are given as mean values of 3-4 consecutive sections per embryo. No significant differences in PrCP area was seen comparing *Gas1*$^{-/-}$ and control embryos using unpaired *t* test. n=9-11 embryos per somite stage and genotype. Scale bars: 50 µm.

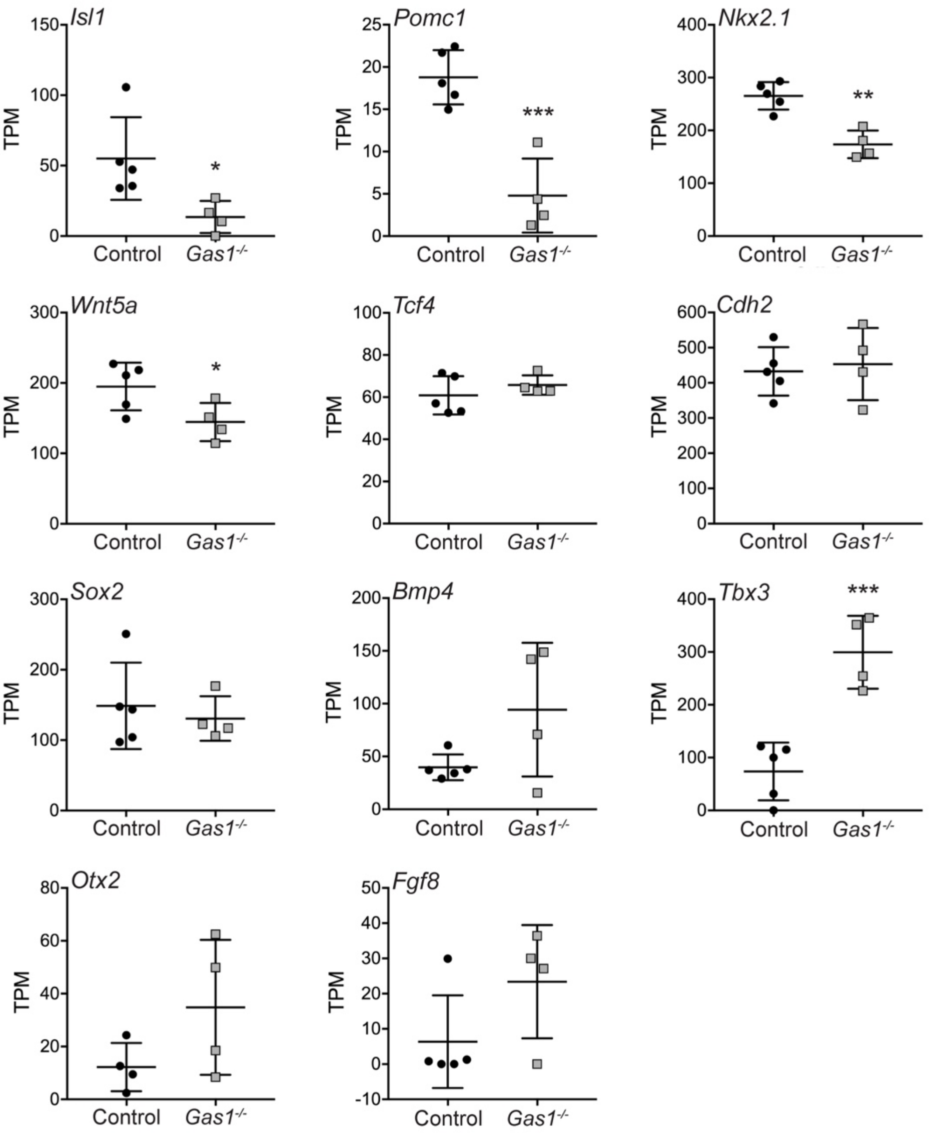

Fig. S2. Global RNA sequencing indicates dysregulated expression of SHH target genes in the rostral ventral forebrain midline of *Gas1*$^{-/-}$ embryos.

RNA expression data for the indicated SHH target genes in the isolated rostral ventral forebrain midline of control and *Gas1*$^{-/-}$ embryos at E10.0 are shown. Levels are given as transcripts per million (TPM). n=5 (Control) and n=4 (*Gas1*$^{-/-}$) samples of 5 embryos per replicate pool. Significant differences were determined using unpaired *t*-test. * $p < 0.05$, ** $p < 0.01$, *** $p < 0.001$.

Development • Supplementary information

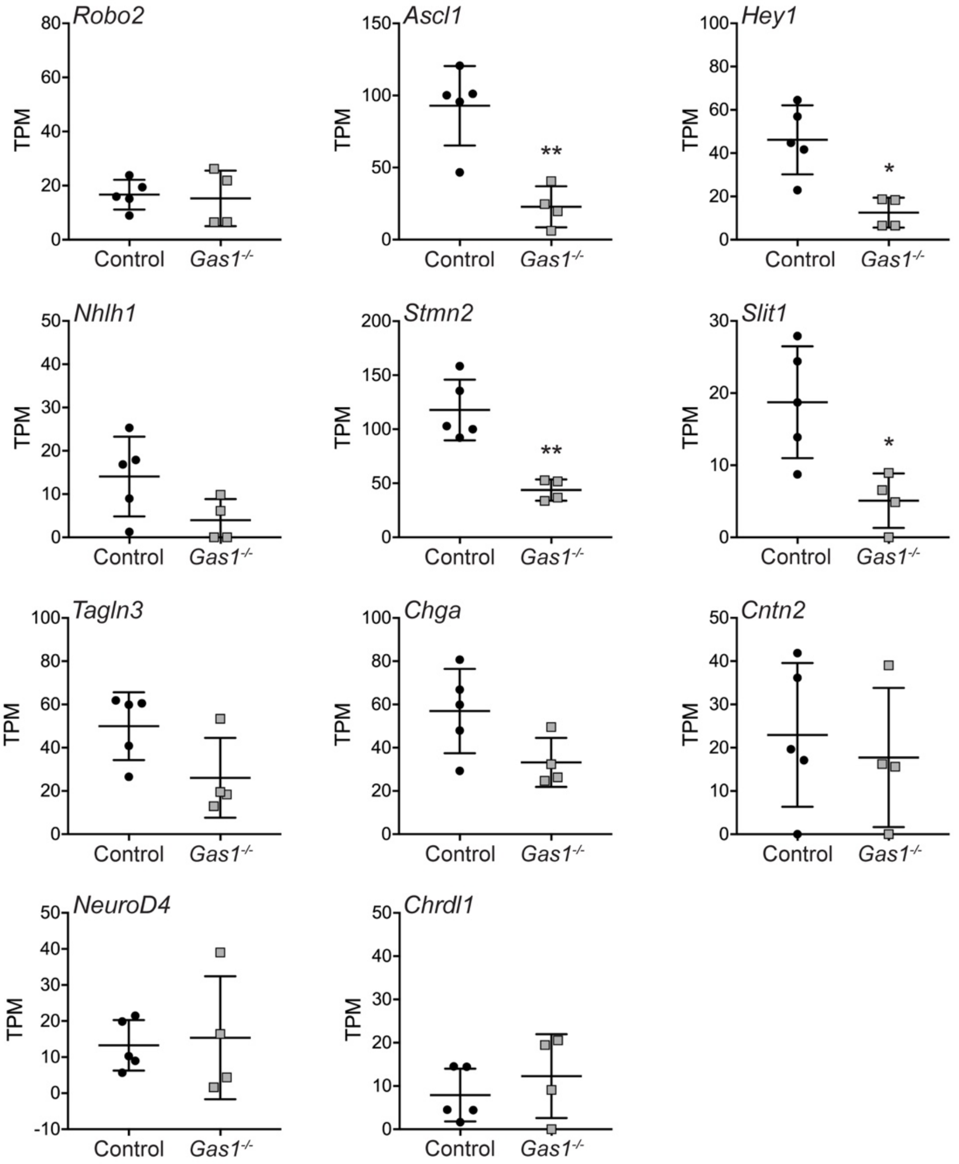

Fig. S3. Global RNA sequencing indicates dysregulated expression of NOTCH target genes in the rostral ventral forebrain midline of *Gas1$^{-/-}$* embryos. RNA expression data for the indicated **NOTCH** target genes in the isolated rostral ventral forebrain midline of control and *Gas1$^{-/-}$* embryos at E10 are shown. Levels are given as transcripts per million (TPM). n=5 (Control) and n=4 (*Gas1$^{-/-}$*) replicate pools with 5 embryos per pool. Significant differences were determined using unpaired *t*-test. * $p < 0.05$, ** $p < 0.01$.

Development • Supplementary information

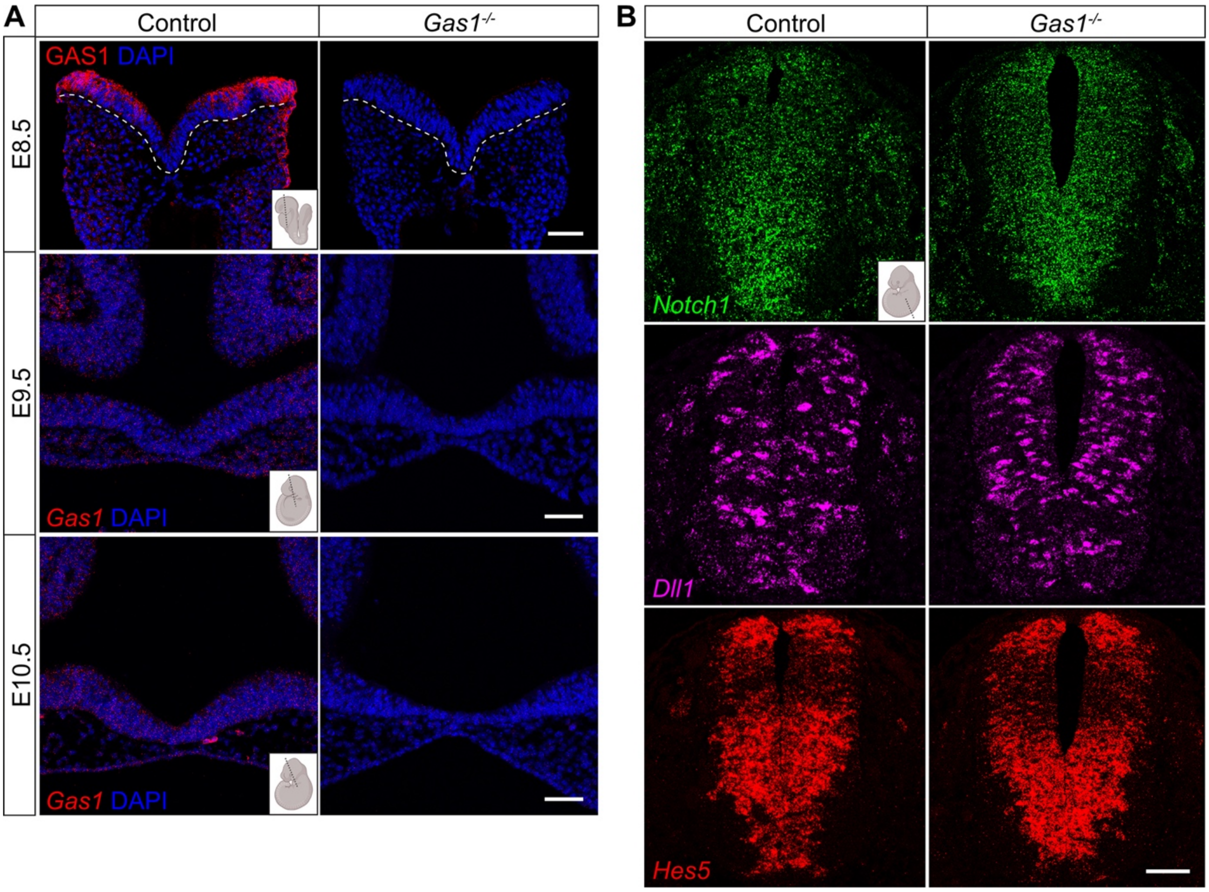

Fig. S4. ***Gas1*** **deficiency does not impact NOTCH activity in the caudal neural tube.** (**A**) Immunodetection of GAS1 protein (upper panel, red) and *Gas1* transcripts (middle and lower panels, red) in the rostral neuroepithelium of control but not *Gas1*$^{-/-}$ embryos at the indicated stages of development. Nuclei are counterstained with DAPI. In the E8.5 image, dotted lines indicate the rostral neuroepithelium. (**B**) Detection of *Notch1* (green; upper panels), *Dll1* (magenta; middle panels), and *Hes5* (red; lower panels) transcripts on coronal sections of E10.5 spinal cord using FISH. Transcript levels in *Gas1*$^{-/-}$ embryos are similar to those in controls. Scale bars: 50 µm. n=3 embryos per somite stage and genotype. Insets demonstrate plane of sections (adapted from BioRender.com).

Development • Supplementary information

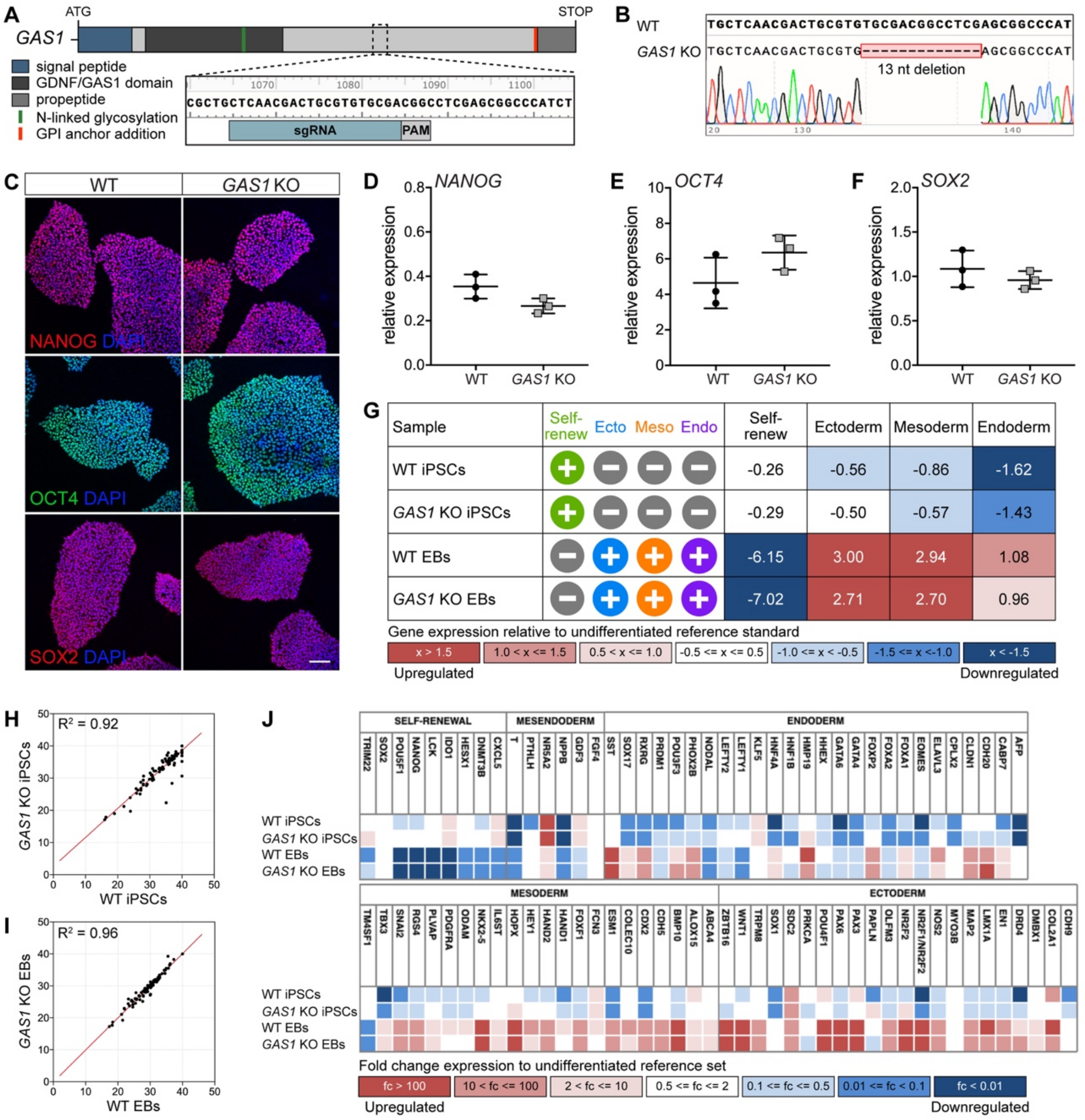

Development • Supplementary information

Fig. S5. iPSC-derived neuroepithelial cells to model GAS1 function in the RDVM

(**A**) CRISPR/Cas9 strategy for disrupting GAS1. Structural organization of human GAS1 indicating the coding sequence targeted by the single guide (sg) RNA. Nucleotide numbers according to human GAS1 sequence NM_002048.3. PAM, protospacer adjacent motif. (**B**) Disruption of the GAS1 coding sequence by a 13 nucleotides long deletion in iPSC line GAS1KO. The respective wild-type *GAS1* sequence is shown above (WT). (**C**) Immunofluorescence detection of pluripotency markers NANOG, OCT4 and SOX2 in WT and *GAS1* KO iPSC lines. Nuclei were counterstained with DAPI. Scale bar: 50 µm. (**D-F**) qRT-PCR of relative transcript levels for *NANOG* (D), *OCT4* (E), and *SOX2* (F) in WT and *GAS1* KO iPSCs. Levels in (D-F) are given as CT values normalized to transcript levels of *GAPDH* ($2^{-\Delta CT}$ ± standard derivation (SD). No statistically significant differences were seen comparing genotypes (unpaired *t* test, n=3 biological replicates). (**G-J**) TaqMan Scorecard analysis of WT and *GAS1* KO iPSCs and spontaneously differentiated embryoid bodies (EBs) after 14 days, respectively. (G) WT and *GAS1* KO iPSCs and EBs showed similar scores for self-renewal, ectoderm, mesoderm and endoderm differentiation compared to the expression profile of the reference standard. EBs downregulated self-renewal genes indicated by (-), while upregulating ectoderm, mesoderm and endoderm genes as indicated by (+). (H, I) Correlation plots depict comparable expression rates of the 96 analyzed genes between iPSCs (H) and EBs (I) of different genotypes. Corresponding correlation coefficients (R^2) are shown in the upper left corner. (J) Heatmaps of the analyzed genes related to self-renewal, mesendoderm, endoderm, mesoderm and ectoderm fate. Values and colors correlate to the fold changes (FC) of each gene relative to the undifferentiated reference set.

Development • Supplementary information

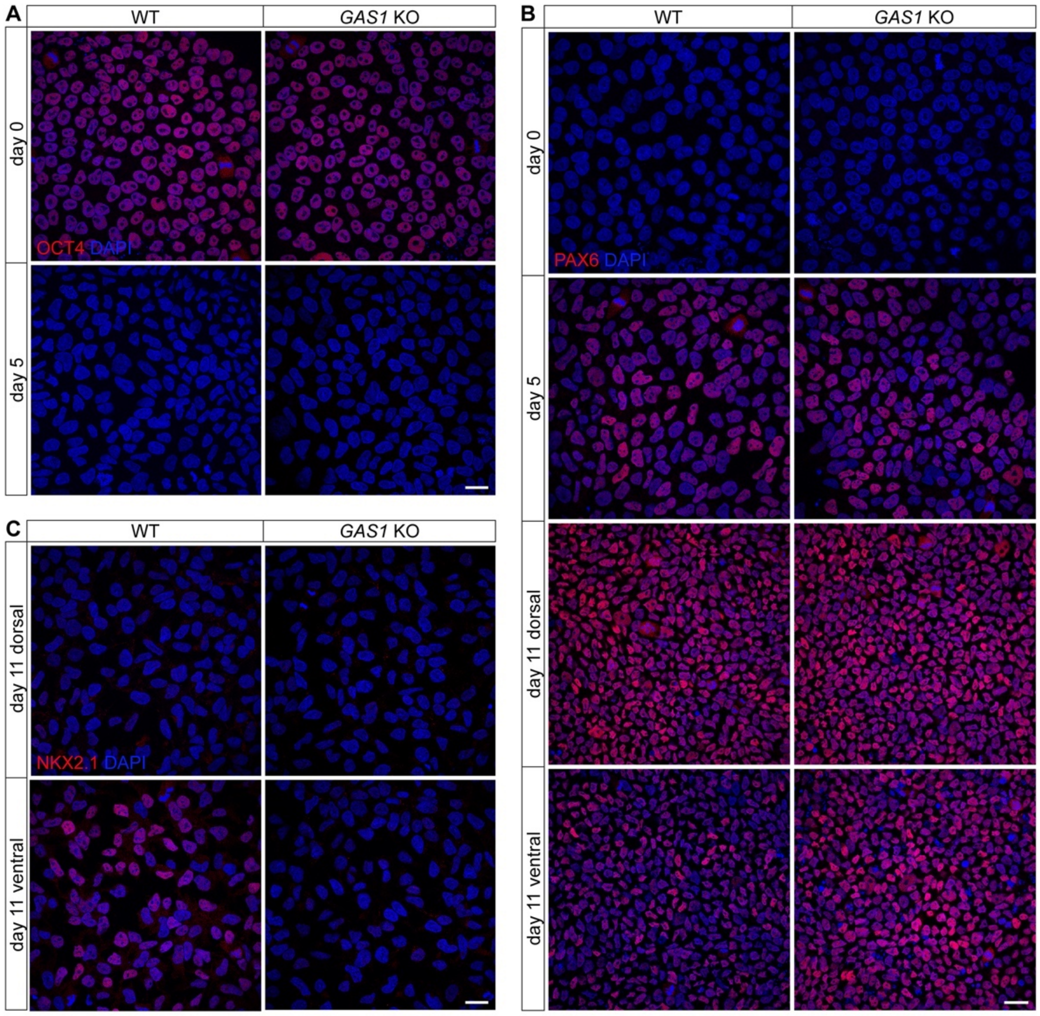

Fig. S6. *GAS1* KO iPSCs fail to induce a SHH-dependent ventral neuroepithelial cell fate.

(**A**) WT and *GAS1* KO iPSCs (day 0), and day 5 NPCs derived thereof, were stained for multipotency marker OCT4 (red). (**B**) Immunodetection of PAX6 (red) in WT and *GAS1* KO iPSCs at the indicated timepoints of neuroepithelial differentiation. The dorsal marker PAX6 is downregulated in WT, but not in *GAS1* KO NPCs, upon SHH-dependent ventral cell fate induction (panel day 11 ventral). (**C**) Immunodetection of NKX2.1 (red) in WT and *GAS1* KO NPCs at day 11 of dorsal or ventral neuroepithelial differentiation. The ventral marker NKX2.1 is upregulated in WT, but not in *GAS1* KO NPCs, upon SHH-induced ventralization. Nuclei in (A-C) were counterstained with DAPI. Scale bars: 25 μm.

Development • Supplementary information

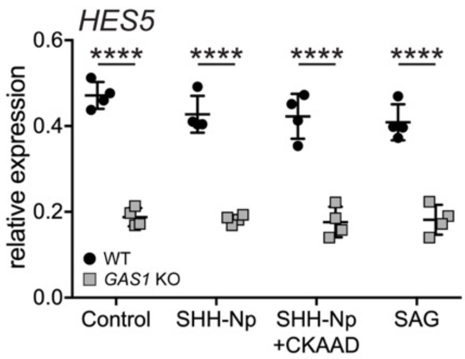

Fig. S7. Loss of SHH signaling does not impact *HES5* expression in NPCs.

Relative transcript levels of *HES5* were determined by qRT-PCR in NPCs at day 8-10 of differentiation. Cells had been treated overnight with control medium, or with medium containing 200 nM smoothened agonist (SAG) or SHH-Np, in the absence or presence of 50 nM cyclopamine-KAAD (CKAAD). n=4 biological replicates per genotype and condition. Levels are given as CT values normalized to transcript levels of *GAPDH* ($2^{-\Delta CT}$ ± standard derivation (SD). Statistical analyses were performed by two-way ANOVA with Bonferroni post hoc test. **** $p < 0.0001$.

Development • Supplementary information

Table S1. Expression values of differentially expressed genes related to the gene ontology term "smoothened signaling pathway" comparing *Gas1*$^{-/-}$ with control embryos

MGI symbol	log2 fold change	q value
Kif7	-8.515	3.794E-04
Sin3a	-7.752	7.008E-03
Nme7	-5.377	3.881E-02
Nkx2.2	-4.422	2.636E-03
Hes5	-3.788	1.350E-02
Ptch1	-1.957	4.501E-02
Shh	-1.825	8.659E-06
Scube1	2.288	1.404E-10
Fgf10	3.054	3.467E-16

Table S2. Expression values of differentially expressed genes related to the gene ontology term "NOTCH signaling pathway" in *Gas1*$^{-/-}$ embryos as compared to controls

MGI symbol	log2 fold change	q value
Tcf3	-22.727	1.566E-09
Sin3a	-7.752	7.008E-03
Traf7	-7.743	3.745E-07
Hes5	-3.788	1.350E-02
Dll1	-3.376	2.040E-05
Mfng	-2.859	6.735E-04
Dll3	-2.619	4.743E-02
Nrarp	-2.011	1.851E-02
Notch1	-1.694	4.825E-04
Fat4	2.063	8.931E-09
Fgf10	3.054	3.467E-16
Tbx2	3.426	1.114E-05

Table S3. Differentially expressed genes (DEGs) comparing the microdissected rostral ventral neuroepithelium from E10.0 *Gas1*$^{+/+}$ and *Gas1*$^{-/-}$ embryos. Fold change in transcript levels are given as *Gas1*$^{-/-}$ compared with *Gas1*$^{+/+}$ embryos. q values represent *P*-values corrected for multiple testing using the Benjamini-Hochberg procedure.

Click here to download Table S3

SUPPLEMENTARY REFERENCES

Carbon, S., Ireland, A., Mungall, C. J., Shu, S., Marshall, B., Lewis, S., Ami, G. O. H. and Web Presence Working, G. (2009). AmiGO: online access to ontology and annotation data. *Bioinformatics* **25**, 288-289.

Flemming, J., Marczenke, M., Rudolph, I. M., Nielsen, R., Storm, T., Erik, I. C., Diecke, S., Emma, F. and Willnow, T. E. (2020). Induced pluripotent stem cell-based disease modeling identifies ligand-induced decay of megalin as a cause of Donnai-Barrow syndrome. *Kidney Int* **98**, 159-167.

Kamburov, A., Pentchev, K., Galicka, H., Wierling, C., Lehrach, H. and Herwig, R. (2011). ConsensusPathDB: toward a more complete picture of cell biology. *Nucleic Acids Res* **39**, D712-717.

Kamburov, A., Wierling, C., Lehrach, H. and Herwig, R. (2009). ConsensusPathDB--a database for integrating human functional interaction networks. *Nucleic Acids Res* **37**, D623-628.

Love, M. I., Huber, W. and Anders, S. (2014). Moderated estimation of fold change and dispersion for RNA-seq data with DESeq2. *Genome Biol* **15**, 550.

Patro, R., Duggal, G., Love, M. I., Irizarry, R. A. and Kingsford, C. (2017). Salmon provides fast and bias-aware quantification of transcript expression. *Nat Methods* **14**, 417-419.

Tsankov, A. M., Akopian, V., Pop, R., Chetty, S., Gifford, C. A., Daheron, L., Tsankova, N. M. and Meissner, A. (2015). A qPCR ScoreCard quantifies the differentiation potential of human pluripotent stem cells. *Nat Biotechnol* **33**, 1182-1192.

Development • Supplementary information

SUPPLEMENTARY MATERIALS AND METHODS

In situ hybridization and immunohistology on mouse tissue

The plasmid for generating the *in situ* hybridization (ISH) probe targeting *Shh* was kindly provided by A. P. McMahon (University of Southern California, Los Angeles). ISH probes detecting *Hes5* and *Nkx2.1* were generated in house targeting basepairs 573-1271 for *Hes5* (reference NM_010419.3) and basepairs 2032-2813 for *Nkx2.1* (reference NM_009385.2). Immunofluorescence staining was carried out using rabbit anti-SHH (1:50, sc-9024, Santa Cruz Biotechnology) or goat anti-GAS1(1:50, AF2644, R&D Systems), respectively, and secondary antibodies conjugated with Alexa Fluor fluorophores (1:1000, Invitrogen). RNAscope fluorescent ISH (FISH) on 6 µm paraffin sections was performed according to manufacturer's protocols using the Multiplex Fluorescent Reagent Kit v2 (ACD) with standard conditions for manual target retrieval and Protease Plus treatment. Following probes from ACD were used: *Shh* (3143661) or *Shh*-C2 (3143661-C2), *Gli1* (311011) or *Gli1*-C2 (311011-C2), *Notch1*-C2 (404641-C2), *Dll1*-C3 (425071-C3), *Hes5* (400991) or *Hes5*-C2 (400991-C2). Bound probes were visualized using Opal dyes 520 (FP1487001KT), 570 (FP1488001KT) or 620 (FP1495001KT, all from Akoya Biosciences) at a dilution of 1:1500.

Microdissection and bulk RNAseq

For laser capture microdissection (LCM), E10.0 embryos (30-33 somites) were dissected on ice and fixed in 2% paraformaldehyde (PFA) for two hours at 4°C. After washing with PBS twice, embryos were infiltrated with 30% sucrose in PBS for two hours at 4°C, sequentially incubated with 25%, 50%, and 75% Tissue Tek O.C.T. compound (Sakura) in 30% sucrose solution for 30 minutes at 4°C each before embedding in Tissue Tek O.C.T. Coronal 12 µm cryosections were collected on membrane slides (MembraneSlide NF 1.0 PEN, 415190-

Development • Supplementary information

9081-000, Zeiss), shortly washed in water, and dehydrated in cold 70% and 100% ethanol. Dried sections were stored in membrane slide chambers at -80°C. Before microdissection, slides were gradually chilled to room temperature (RT) by incubating at -20°C, 4°C, and RT for 20 minutes each. LCM was performed by using the Zeiss Axio Observer Z1. Dissected rostral ventral forebrain midlines were collected in AdhesiveCap 500 tubes (415190-9201-000, Zeiss), directly lysed in RNA lysis buffer, shortly stored on dry ice, and then transferred to -80°C. For processing, samples were thawed on ice and RNA was isolated using the RNeasy FFPE Kit (Qiagen) according to the manufacturer's manual. RNA integrity and yield were assessed using the Bioanalyzer 2100 (Agilent Technologies) and Qubit fluorometric quantification (Thermo Fisher Scientific), respectively. RNA with RNA integrity number (RIN) > 6.5 was used for cDNA library preparation using the SMARTer Stranded Total RNA-Seq Pico Kit (Takara), and libraries were sequenced in a 2 x 75 bp paired end run on an Illumina HiSeq 4000 system with 20 million reads per sample. The analysis involved five replicates for control and four replicates for *Gas1*$^{-/-}$ embryos. Each replicate consisted of a pool of five embryos of the respective genotype.

Salmon v0.12.0 (Patro et al., 2017) was used to quantify the expression of transcripts against the Ensembl release 94 of the *Mus musculus* transcriptome. The multiple forms of noncoding RNA present in the total RNA sequencing were removed from the GTF file provided by the same Ensembl release. Only "protein_coding" and lincRNA transcript types were kept. Transcripts quantified by Salmon but not present in the filtered GTF file were removed for the downstream analysis. DESeq2 R package (Love et al., 2014) was used with default parameters to find the differentially expressed genes (DEGs). Transcripts with less than 5 read counts were filtered and p-values were corrected for multiple testing using the Benjamini-Hochberg procedure. DEGs recorded in at least two genotypic replicates were subjected to gene ontology (GO) term enrichment analysis using AmiGO (Carbon et al.,

Development • Supplementary information

2009) and ConsensusPathDB (Kamburov et al., 2011; Kamburov et al., 2009), over-representation analysis and default parameters, while only GO terms with q-value ≤ 0.05 were considered. Heatmaps and PCA were generated using the heatmap.2 and prcomp function from the ggplot2 library in RStudio, respectively.

The RNA-seq data have been deposited in the ArrayExpress database at EMBL-EBI under accession number E-MTAB-10207.

Scorecard assay

Pluripotency and the ability of generating all three germ layers were assessed by TaqMan Scorecard Assay (Applied Biosystems) on spontaneously differentiated embryoid bodies (EB) (Tsankov et al., 2015). For EB formation, iPSCs were dissociated with Accutase, seeded into Nucleon Sphera 96-well U-shaped-bottom plates (Thermo Fisher Scientific) at a density of 8000 cells/well in E8 medium supplemented with 10 µg/ml Y27632, and centrifuged for 3 minutes at 200 g. Next day, the medium was replaced by DMEM/F12 supplemented with 10% KnockOut Serum Replacement and 1% penicillin-streptomycin (Gibco). The medium was changed every 3 days. iPSCs (day 0) and EBs (day 14, pool of 20 EBs per genotype) were subjected to RNA isolation followed by cDNA reverse transcription using the RNeasy Mini (Qiagen) and High-Capacity RNA-to-cDNA Kit (Applied Biosystems), respectively, before performing the Scorecard assay according to the manufacturer's protocols. Gene expression data were analyzed via the web-based hPSC Scorecard Analysis Software (Thermo Fisher Scientific).

Generation of neural progenitor cells

The protocol for differentiating WT and *GAS1* KO cells into NPCs was published before (Flemming et al., 2020). Briefly, iPSCs were dissociated with Accutase and 20.000 cells/cm^2

Development • Supplementary information

were plated on Matrigel-coated plates in E8 medium supplemented with 10 µg/ml Y27632 for 24 hours. Cells were allowed to grow for 3 days in E8 medium until they were nearly confluent, after which the medium was changed to N2B27 differentiation medium containing 100 ng/ml noggin (6057-NG, R&D Systems), 200 nM dorsomorphin (sc-361173A, Santa Cruz Biotechnology) inhibiting BMP signaling and 10 µM SB431542 (Cay13031, Cayman Chemical) for the inhibition of TGFβ signaling. The medium was changed daily. For ventral-like NPC induction, the medium was replaced by N2B27 containing noggin, dorsomorphin, and 200 ng/ml SHH at day 5 of differentiation, and cells were grown for up to 6 more days.

Immunofluorescence staining and immunohistology of iPSCs/NPCs

Cells were washed with PBS to remove cell debris and fixed in 4% PFA for 15 minutes at RT. After washing with PBS, cells were permeabilized in blocking buffer containing 5% BSA and 0.5% Triton X100 for 1 hour at RT before incubating with primary antibodies diluted in blocking buffer at 4°C overnight. After washing in TBS with 1% Triton X-100 for 30 minutes and subsequently in TBS with 0.1% Tween 20, cells were incubated with secondary antibodies conjugated with Alexa Fluor fluorophores (Invitrogen) diluted 1:1000 in blocking buffer for 1-2 hours at RT. Cells were counterstained with DAPI and mounted with ProLong Gold Antifade Mountant (Life Technologies). Following primary antibodies were used: sheep anti-DLL1 (1:100, AF5026, R&D Systems), goat anti-GAS1 (1:50, AF2636, R&D Systems), goat anti-NANOG (1:100, AF1997, R&D Systems), rabbit anti-NKX2.1 (1:40, MAB94581, R&D Systems), sheep anti-NOTCH1 (1:200, AF5267, R&D Systems), rabbit anti-PAX6 (1:200, 901301, BioLegend), rabbit anti-OCT4 (1:400, sc-5279, Santa Cruz Biotechnology) and rabbit anti-SOX2 (1:100, ab97959, Abcam).

RNAscope FISH of *HES5* transcripts was performed using the Multiplex Fluorescent Reagent Kit v2 (ACD) according to manufacturer's protocol and the technical note

"RNAscope Assay for Adherent Cells Cultured on Coverslips". Bound human *HES5* probe (521391) was visualized using Opal dye 570 (FP1488001KT) at a dilution of 1:1000.

Proximity ligation assay

Proximity ligation assay (PLA) was performed according to the manufacturer's instructions (Duolink PLA Fluorescence). PFA-fixed NPCs at differentiation day 7-9 were incubated with directly conjugated primary antibodies at a dilution of 1:100 generated with Duolink In Situ Probemaker PLUS and MINUS Kits. PLA signals were visualized using the Duolink In Situ Detection Reagent Kit Orange (all Sigma-Aldrich). Following primary antibodies were used: goat anti-GAS1 (AF2636, R&D Systems), sheep anti-NOTCH1 (AF5267, R&D Systems) and rabbi anti-PTCH1 (homemade).

Quantitative real-time-PCR

Total RNA was isolated using the RNeasy Mini Kit (Qiagen) according to the manufacturer's instructions with additionally treating the samples with RNase-free DNAse I (Qiagen) for 15 minutes at RT. One µg total RNA was reverse transcribed using the High-Capacity RNA-to-cDNA Kit (Applied Biosystems). The resulting cDNA was amplified using TaqMan oligonucleotide probes and the TaqMan Gene Expression Master Mix on a 7900HT Fast Real time PCR System and the Sequence detection system V2.4 (all Applied Biosystems). Results were analyzed using the comparative cycle threshold (CT) method. TaqMan probes from Applied Biosystems were *DLL1* (Hs00194509_m1), *DLX2* (Hs00269993_m1), *FOXG1* (Hs01850784_s1), *GAPDH* (Hs02758991_g1), *GAS1* (Hs00266715_s1), *GLI1* (Hs00171790_m1), *GLI3* (Hs00609233_m1), *HES5* (Hs01387463_g1), *LHX6* (Hs01030943_m1), *NANOG* (Hs02387400_g1), *NKX2.1* (Hs00968940_m1), *NKX2.2*

Development • Supplementary information

(Hs00159616_m1), *NOTCH1* (Hs01062014_m1), *OCT4* (Hs00999632_g1), *PAX6* (Hs00240871_m1) and *SOX2* (Hs01053049_s1).

Co-immunoprecipitation

NPCs were differentiated until day 6, seeded onto Matrigel-coated 6-well-plates at a density of 250000 cells/well, and transfected with pCIG-hGAS1-IRES-GFP (kindly provided by F. Charron, McGill University, Canada) using Lipofectamine2000 (Invitrogen) according to manufacturer's protocol. 48 hours after transfection, cells were fixed in 1% formaldehyde (Thermo Fisher Scientific) for 5 minutes at RT and quenched with cold 1.25 M glycine. Co-immunoprecipitation (Co-IP) was performed using the Pierce Crosslink Magnetic IP/Co-IP Kit (Thermo Fisher Scientific). For each reaction, 10 µg/ml goat anti-GAS1 antibody (AF2636, R&D Systems) or a non-immmune goat IgG control (01-6202, Invitrogen) were bound to 50 µl magnetic beads for 1 hour and crosslinked for 30 minutes. Cells were lysed with IP Lysis/Wash Buffer supplemented with cOmplete Protease Inhibitor Cocktail (Roche) for 1-2 hours on ice on a vertical shaker. The Pierce BCA Protein Assay Kit (Thermo Fisher Scientific) was used to determine protein concentration in cell lysates. Thirty µg protein lysate was used as input control and 200-300 µg protein lysate was incubated with crosslinked beads at 4°C overnight. Elution was performed for 30 minutes at RT on a rotator. Samples were incubated with Novex Tris-Glycine SDS Sample buffer (Invitrogen) supplemented with 2.5% 2-Mercaptoethanol for 5 minutes at 60°C, before subjecting to protein analysis by western blotting.

Western blot analysis

Protein expression in cells was evaluated by western blot analysis using standard procedures. Primary antibodies used for immunodetection were goat anti-GAS1 (1:500, AF2636, R&D

Development • Supplementary information

Systems), mouse anti-HA.11 (1:1000, 901513, BioLegend), rabbit anti-NOTCH1 (1:1000, 4380, Cell Signaling Technology), rabbit anti-cleaved NOTCH1 (1:500, 4147, Cell Signaling Technology) and mouse anti-tubulin (1:1000, CP06, Calbiochem). Primary antibodies were detected using secondary antibodies conjugated with horseradish peroxidase (diluted 1:1500; Sigma-Aldrich) and SuperSignal West Femto Maximum Sensitivity Substrate (Thermo Fisher Scientific). Protein levels were quantified by densitometric scanning of western blots using the Odyssey Fc Imaging System and Image Studio Lite Software (LI-COR Biosciences).

Lentivirus production

For producing NICD or GFP overexpressing lentivirus particles, HEK293TN cells were cultivated on 0.0025% poly-L-Lysin (Sigma-Aldrich) coated culture flasks in Opti-MEM (Gibco). Cells were transfected with lentivirus envelope and packaging plasmids pMD2.D, pMDLg/pRRE and pRSV-Rev (Addgene plasmids #12259, #12251 and #12253, respectively, and the control plasmid EF.PGK.GFP (Addgene plasmid #17618) or the NICD expressing plasmid NOTCH1 intracellular domain-pcw107-V5 (Addgene plasmid #64622) using Lipofectamine2000 according to manufacturer's instructions in Opti-MEM supplemented with 25 µM chloroquine (Sigma-Aldrich). After 6 hours, the medium was changed to Opti-MEM supplemented with 10 µM sodium butyrate (Sigma-Aldrich). Cells were further cultivated for 2-3 days and the medium replaced and collected every day. The collected medium was centrifuged for 15 minutes at 500 g at 4°C to remove cell debris and every 3 volumes of lentivirus containing supernatant was incubated with 1 volume of cold Lenti-X Concentrator (Clontech Laboratories) on ice at 4°C overnight. The mixture was centrifuged at 4°C for 45 minutes at 1500 g and lentiviral pellets were resuspended in 1:100 of the original volume using cold and sterile PBS. Lentiviral particle containing solutions were aliquoted and stored at -80°C.

Development • Supplementary information
