# Supplementary material for: GAS1 is required for NOTCH-dependent facilitation of SHH signaling in the ventral forebrain neuroepithelium
Source: Development. 2021 Nov 11;148(21):dev200080. doi: 10.1242/dev.200080 (PMC8627604; doi:10.1242/dev.200080)
Supplement: Reviewer comments [file develop-148-200080_review_history.pdf]

GAS1 is required for NOTCH-dependent facilitation of SHH signaling in the ventral forebrain neuroepithelium

Maike Marczenke, Daniele Yumi Sunaga-Franze, Oliver Popp, Irene W. Althaus, Sascha Sauer, Philipp Mertins, Annabel Christ, Benjamin L. Allen and Thomas E. Willnow
DOI: 10.1242/dev.200080

Editor: James Briscoe

Review timeline

Original submission:	5 August 2021
Editorial decision:	3 September 2021
First revision received:	22 September 2021
Editorial decision:	23 September 2021
Second revision received:	24 September 2021
Accepted:	27 September 2021

Original submission

First decision letter

MS ID#: DEVELOP/2021/200080

MS TITLE: GAS1 is required for Notch-dependent facilitation of SHH signaling in neuroepithelial cells

AUTHORS: Maike Marczenke, Daniele Yumi Sunaga-Franze, Oliver Popp, Irene Althaus, Sascha Sauer, Philipp Mertins, Annabel Christ, Benjamin L. Allen, and Thomas Willnow

I have now received all the referees reports on the above manuscript, and have reached a decision. The referees' comments are appended below, or you can access them online: please go to BenchPress and click on the 'Manuscripts with Decisions' queue in the Author Area.

The overall evaluation is positive and we would like to publish a revised manuscript in Development, provided that the referees' comments can be satisfactorily addressed. Please attend to all of the reviewers' comments in your revised manuscript and detail them in your point-by-point response. If you do not agree with any of their criticisms or suggestions explain clearly why this is so.

We are aware that you may currently be unable to access the lab to undertake experimental revisions. If it would be helpful, we encourage you to contact us to discuss your revision in greater detail. Please send us a point-by-point response indicating where you are able to address concerns raised (either experimentally or by changes to the text) and where you will not be able to do so within the normal timeframe of a revision. We will then provide further guidance. Please also note that we are happy to extend revision timeframes as necessary.

Reviewer 1

Advance summary and potential significance to field

This manuscript by Marczenke et al provides evidence that GAS1, a known co-receptor for Hh signaling, also plays a novel role in directly regulating Notch signaling. This work adds to a growing body of work on the crosstalk between Notch and Hh signaling.

© 2021. Published by The Company of Biologists under the terms of the Creative Commons Attribution License (https://creativecommons.org/licenses/by/4.0/).

Comments for the author

The authors have done an excellent job in revising the manuscript and addressing my concerns. I recommend publication of this manuscript in Development.

Minor comments:
1. Line 111-113, “Importantly, our findings uncovered that GAS1 is not essential for SHH signaling in this tissue, but for persistence of its activity domain, initially established in the absence of this receptor.” This sentence is confusing and needs to be revised.

2. Line 387, the reference of Huang et al., 2012 is incorrect and should be removed from this sentence.

3. Fig 8F, SMO should be a transmembrane protein in the model.

Reviewer 2

Advance summary and potential significance to field

Through bulk RNAseq of rostral ventral neural tube and in vivo expression analyses, the authors provide evidence for a role for Gas1 in Notch signalling. Next, iPSC lines, wild-type or genetically deficient for GAS1, are established and subject to differentiation conditions (dorsalising and ventralising (SHH)). In GAS1 mutant lines, SHH fails to repress PAX6 and GLI3, or to upregulate NKX2-1. HES5 levels are reduced in GAS1 mutant lines, in both dorsalising and ventralising conditions. Biochemical studies on the iPSC lines indicate that GAS1 and NOTCH may physically interact, and that membrane-tethered GAS1 promotes NOTCH signalling. The authors then bring these studies together, to suggest that GAS1 is required for Notch-dependent facilitation of Shh signalling in forebrain development. This is nicely shown in an ex vivo assay of Gas1 mutant neuroepithelium, where a subset of explant express Shh after ectopic expression of NICD.

The finding that Gas1 is required for Notch-dependent facilitation of Shh signalling in the forebrain is novel, timely and extremely interesting

Comments for the author

I would just like to thank the authors for the extensive revisions, which have much-improved the manuscript. The vast majority of my concerns have been addressed. Figure 8, in particular, is so much more cconvincing than previous Figure 4.

Reviewer 3

Advance summary and potential significance to field

The authors provide evidence that Gas1, a co-receptor for Hedgehog signalling, plays a specific role during early forebrain development. This function would be Shh signalling-independent and involve Notch pathway.

They have used a Gas1-deficient mouse model to show that some Notch components are differentially expressed in the rostral diencephalon ventral midline in absence of Gas1. Then, they generated iPSC lines genetically deficient for Gas1 and demonstrated that this gene promotes Notch signalling. This observation was supported by ex vivo cultures of cephalic explants treated with lentiviral constructs to induce Notch signalling.

This study contributes to our better understanding of vertebrate brain development as this shh-independent function of Gas1 during forebrain development is novel. It also shows the importance of precisely regulating the dosage of Shh specifically in the developing ventral forebrain. It also provides a new molecular mechanism implicated in a complex rare disease, holoprosencephaly.

© 2021. Published by The Company of Biologists under the terms of the Creative Commons Attribution License (https://creativecommons.org/licenses/by/4.0/).

Comments for the author

- An important finding is that this Notch/Gas 1 interaction would be diencephalon ventral-specific; the authors have shown that Gas1 deficiency does not impact Notch activity in the caudal neural tube (Fig.S4).
This should be emphasised in the title because "neuroepithelial cells " used in the title of this manuscript is too general.
- Page 5, Line 106: description of the figure 1 should be nuanced as at E10.5 Shh transcript in the ventral neuroepithelium was only decreased whereas Shh protein almost completely lost (as far as I can see from the figure).
- I am puzzled that the most important genes that are Nkx2.1, Tbx2... are not present in the heatmap of differentially expressed genes in Fig2; they are relegated to the supplementary data (S2).
Furthermore, the list of 324 genes that are differentially expressed should be published in the supplementary data; it will surely be of interest to many readers.
- In the context of direct interaction between Gas1 and Notch, it would be important to show that Gas1 is expressed in the rostral diencephalon ventral midline, as are Notch1, Dll1 and Hes5 at E9.5 and E10.5 (Fig3).
In this the manuscript only Gas1 expression at E8.5 (Fig S4) is shown and this expression is not detectable in the midline. Gas1 expression in control at E9.5 and E10.5 should be included to a revised manuscript.
- From E9.0, Shh expression is totally NOTCH-dependent in the rostral diencephalon ventral midline (as it has been demonstrated by Hamdi-Roze, 2020). These mouse models demonstrated that Notch deficiency fully contributes to the loss of responsiveness to Shh in the rostral diencephalon ventral midline. In the light of the results presented here, to what extent Gas1 would also activate the Shh pathway directly? This needs to be discussed?
-To my knowledge there is no standard nomenclature for writing " the name of the gene" of a pathway.
However in a manuscript, it must be standardised. Either use uppercase (SHH and NOTCH signalling/pathway) or use lowercase (Shh and Notch signalling/pathway).
- The mouse model ShhCREEGFP is not described in the supplementary methods SUP FIG 5: n=1 for each condition, n=2 would have been better.

Line 310: pattering instead of patterning
Line 353: Ware 2016 instead of Ware 2014.
Line 391: NOTCH1 should be replaced by NOTCH.

First revision

Author response to reviewers' comments

COMMENTS TO REVIEWER 1:
"The authors have done an excellent job in revising the manuscript and addressing my concerns. I recommend publication of this manuscript in Development."

> We very much appreciate this reviewer's enthusiastic evaluation of our revisions.

Minor comments:
1. Line 111-113, "Importantly, our findings uncovered that GAS1 is not essential for SHH signaling in this tissue, but for persistence of its activity domain, initially established in the absence of this receptor." This sentence is confusing and needs to be revised.

> We have revised this sentence for clarity. It now reads "Importantly, our findings uncovered that GAS1 is not essential for SHH signaling in this tissue. Rather it promotes persistence of this SHH activity domain, initially established in the absence of this receptor." (lines 113 - 115).

© 2021. Published by The Company of Biologists under the terms of the Creative Commons Attribution License (https://creativecommons.org/licenses/by/4.0/).

2.Line 387, the reference of Huang et al., 2012 is incorrect and should be removed from this sentence.

> We apologize for erroneous citation of this article which has now been removed from this sentence.

3.Fig 8F, SMO should be a transmembrane protein in the model.

> We now have drawn SMO in the schematic in Fig. 8F in a transmembrane configuration.

COMMENTS TO REVIEWER 3

Point 1:
- An important finding is that this Notch/Gas 1 interaction would be diencephalon ventral-specific; the authors have shown that Gas1 deficiency does not impact Notch activity in the caudal neural tube (Fig.S4). This should be emphasised in the title because "neuroepithelial cells " used in the title of this manuscript is too general.

> We now rephrased the title to more clearly reflect the fact that GAS1 action in NOTCH signaling is spatially restricted. The revised title reads: GAS1 is required for NOTCH-dependent facilitation of SHH signaling in the ventral forebrain neuroepithelium.

Point 2:
- Page 5, Line 106: description of the figure 1 should be nuanced as at E10.5 Shh transcript in the ventral neuroepithelium was only decreased whereas Shh protein almost completely lost (as far as I can see from the figure).

> We revised the text to accentuate these nuances. The corresponding text reads: “By contrast, Shh and Gli1 transcripts in the rostral ventral neuroepithelium of Gas1-/- embryos were decreased by E9.5 (Fig. 1C) and SHH protein was completely lost by E10.5, when compared to controls (Fig. 1D). (lines 105 - 107)

Point 3:
- Furthermore, the list of 324 genes that are differentially expressed should be published in the supplementary data; it will surely be of interest to many readers.

> We now provide the list of 324 DEGs in a supplementary excel data file.

Point 4:
- In this the manuscript only Gas1 expression at E8.5 (Fig S4) is shown and this expression is not detectable in the midline. Gas1 expression in control at E9.5 and E10.5 should be included to a revised manuscript.

> New data documenting expression of Gas1 in the ventral forebrain neuroepithelium of wild-type but not GAS1 mutant embryos at E9.5 and E10.5 have now been included in the revised manuscript (Fig. S4A).

Point 5:
- From E9.0, Shh expression is totally NOTCH-dependent in the rostral diencephalon ventral midline (as it has been demonstrated by Hamdi-Roze, 2020). These mouse models demonstrated that Notch deficiency fully contributes to the loss of responsiveness to Shh in the rostral diencephalon ventral midline. In the light of the results presented here, to what extent Gas1 would also activate the Shh pathway directly? This needs to be discussed?

As documented in our study, GAS1 acts through two distinct mechanisms in control of SHH signaling during forebrain development, one mechanism operating through PTCH1 and one operating through NOTCH1 (to increase SHH signal strength). To what extent GAS1 action on PTCH1, rather than on NOTCH1, contributes to SHH signal strength in the forebrain neuroepithelium is difficult to assess in vivo. However, based on quantitative modeling of morphogen action in NPCs, GAS1 mainly operates

© 2021. Published by The Company of Biologists under the terms of the Creative Commons Attribution License (https://creativecommons.org/licenses/by/4.0/).

through NOTCH1, rather than through PTCH1, in promoting SHH action in the early ventral forebrain neuroepithelium.

These above considerations are elaborated on in the discussion section. The corresponding text in the discussion reads "While dissection of Notch-dependent versus NOTCH-independent effects of GAS1 on SHH signaling will be challenging in vivo, iPSC-based modelling of neural progenitor differentiation enables quantitative assessment of the contribution of both pathways to SHH signal strength. In WT NPCs, SHH-Np induced gene expression is largely reduced (GLI1) or even completely abolished (NKX2.1) by blockade of NOTCH using DAPT (Fig. 7B and C). In support of a prominent role for NOTCH in SHH signal strength, SHH signaling defects in GAS1 mutant NPCs can be partially rescued in vitro (Fig. 7E-F) and in cephalic explants ex vivo (Fig. 8D-E) by NICD. Although this experimental approach does not formally rule out a GAS1-independent role for NOTCH in SHH signal transduction, SHH-Np induced expression of GLI1 is comparable in WT and GAS1 KO cells in the presence of DAPT, arguing that a major contribution to SHH signal strength in WT cells stems from the action of GAS1 on NOTCH (Fig. 7B)." (lines 395 – 406).

We trust that these edits provide an accurate and balanced account of our data and the conclusions that can be drawn from these.

Point 6:
-To my knowledge there is no standard nomenclature for writing " the name of the gene" of a pathway. However in a manuscript, it must be standardised. Either use uppercase (SHH and NOTCH signalling/pathway) or use lowercase (Shh and Notch signalling/pathway).

> We apologize for inconsistent use of the nomenclature. We now have unified the terminology throughout the manuscript by using uppercase letters (i.e., SHH and NOTCH signaling).

Point 7:
- The mouse model ShhCREEGFP is not described in the supplementary methods
SUP FIG 5: n=1 for each condition, n=2 would have been better.

> The description of the ShhCre-eGFP mouse line is given in the main method section (line 418).

To address an initial query by reviewer 2 (major point 2), whether SHH may control expression of NOTCH pathway components, we purchased mice from the JAX laboratories carrying a targeted insertion of a Cre-eGFP expression construct in the endogenous murine Shh locus. We set up timed mating of mice heterozygous for the transgene to obtain homozygous embryos genetically deficient for Shh. Unfortunately, from the 4 purchased females, we were only able to obtain one Shh null embryo at the desired age (E8.5). Analysis of this embryo is depicted as supplementary information in Fig. S5 and fully confirms that loss SHH activity does impact expression of NOTCH pathway components.

Reviewer 2 has been content with these data. If n=1 is not acceptable as scientific evidence, we will remove these data from the manuscript. This image does not provide crucial evidence as the fact, that SHH does not control expression of NOTCH pathway components, has also been shown unambiguously in this manuscript in replicate experiments using cultured NPCs (Fig. S8). Thus, the omittance of the mouse data in Fig. S5 will not at all change the conclusions to be drawn from our study.

Point 8:
Line 310: pattering instead of patterning
Line 353: Ware 2016 instead of Ware 2014.
Line 391: NOTCH1 should be replaced by NOTCH.

> We apologize for these typographical errors which have been corrected in the revised manuscript.

© 2021. Published by The Company of Biologists under the terms of the Creative Commons Attribution License (https://creativecommons.org/licenses/by/4.0/).

Second decision letter

MS ID#: DEVELOP/2021/200080

MS TITLE: GAS1 is required for Notch-dependent facilitation of SHH signaling in the ventral forebrain neuroepithelium

AUTHORS: Maike Marczenke, Daniele Yumi Sunaga-Franze, Oliver Popp, Irene Althaus, Sascha Sauer, Philipp Mertins, Annabel Christ, Benjamin L. Allen, and Thomas Willnow

We remain keen to publish a revised manuscript in Development. Thank you for addressing the remaining referees' comments. I agree with you that it would be better to remove the data from the Shh mutant embryo in Fig. S5 as it is n=3D1. Once you have adjusted the manuscript accordingly, I will be happy to accept it.

We are aware that you may currently be unable to access the lab to undertake experimental revisions. If it would be helpful, we encourage you to contact us to discuss your revision in greater detail. Please send us a point-by-point response indicating where you are able to address concerns raised (either experimentally or by changes to the text) and where you will not be able to do so within the normal timeframe of a revision. We will then provide further guidance. Please also note that we are happy to extend revision timeframes as necessary.

Second revision

Author response to reviewers' comments

Supplementary figure 5 (Fig. S5) removed from the manuscript.

Third decision letter

MS ID#: DEVELOP/2021/200080

MS TITLE: GAS1 is required for Notch-dependent facilitation of SHH signaling in the ventral forebrain neuroepithelium

AUTHORS: Maike Marczenke, Daniele Yumi Sunaga-Franze, Oliver Popp, Irene Althaus, Sascha Sauer, Philipp Mertins, Annabel Christ, Benjamin L. Allen, and Thomas Willnow
ARTICLE TYPE: Research Article

I am happy to tell you that your manuscript has been accepted for publication in Development, pending our standard ethics checks.

© 2021. Published by The Company of Biologists under the terms of the Creative Commons Attribution License (https://creativecommons.org/licenses/by/4.0/).
